# A Global *In Vivo Drosophila* RNAi Screen Identifies a Key Role of Ceramide Phosphoethanolamine for Glial Ensheathment of Axons

**DOI:** 10.1371/journal.pgen.1003980

**Published:** 2013-12-12

**Authors:** Aniket Ghosh, Tina Kling, Nicolas Snaidero, Julio L. Sampaio, Andrej Shevchenko, Heribert Gras, Bart Geurten, Martin C. Göpfert, Jörg B. Schulz, Aaron Voigt, Mikael Simons

**Affiliations:** 1 Max Planck Institute for Experimental Medicine, Göttingen, Germany; 2 Department of Neurology, University of Göttingen, Göttingen, Germany; 3 Max Planck Institute of Molecular Cell Biology and Genetics, Dresden, Germany; 4 Department of Cellular Neurobiology, University of Göttingen, Göttingen, Germany; 5 Department of Neurology, University Medical Center, RWTH Aachen, Aachen, Germany; Harvard Medical School, Howard Hughes Medical Institute, United States of America

## Abstract

Glia are of vital importance for all complex nervous system. One of the many functions of glia is to insulate and provide trophic and metabolic support to axons. Here, using glial-specific RNAi knockdown in *Drosophila*, we silenced 6930 conserved genes in adult flies to identify essential genes and pathways. Among our screening hits, metabolic processes were highly represented, and genes involved in carbohydrate and lipid metabolic pathways appeared to be essential in glia. One critical pathway identified was *de novo* ceramide synthesis. Glial knockdown of lace, a subunit of the serine palmitoyltransferase associated with hereditary sensory and autonomic neuropathies in humans, resulted in ensheathment defects of peripheral nerves in *Drosophila*. A genetic dissection study combined with shotgun high-resolution mass spectrometry of lipids showed that levels of ceramide phosphoethanolamine are crucial for axonal ensheathment by glia. A detailed morphological and functional analysis demonstrated that the depletion of ceramide phosphoethanolamine resulted in axonal defasciculation, slowed spike propagation, and failure of wrapping glia to enwrap peripheral axons. Supplementing sphingosine into the diet rescued the neuropathy in flies. Thus, our RNAi study in *Drosophila* identifies a key role of ceramide phosphoethanolamine in wrapping of axons by glia.

## Author Summary

Glia are essential for the function of any nervous system. The number of glia correlates with the complexity of the nervous system. Important functions of glia include maintaining ionic homeostasis, supporting neurotransmission, and insulating axons to speed up nerve conduction. The biomedical relevance of glia is highlighted by an increasing number of neurological diseases, in which glia appear to play an essential role, ranging from neuropathies to schizophrenia. Here, we performed a global *in vivo* glial-specific RNAi screen of evolutionary conserved genes in *Drosophila*. With this approach, we identified 736 candidate genes that resulted in lethality or motor deficits when knocked down specifically in glia. One essential pathway identified was ceramide phosphoethanolamine biosynthesis, which was found to be important for wrapping glia to extend their membrane around axons of the peripheral nerve. Our study illustrates that a large-scale screen in *Drosophila*, in combination with morphological analysis is able to dissect the basic mechanism of neuron-glia communication and identify candidate genes in human neuropathies.

## Introduction

Many of the essential functions of glia such as neurotransmitter metabolism, ion buffering, axon pathfinding, electrical insulation, and trophic support are conserved between vertebrates and invertebrates [1], [2]. Among the various tasks that glia perform, the ensheathment of axons is one function that is of high clinical relevance as there are several human diseases in which the ensheathing membrane is broken down. In vertebrates, the electrical insulation is executed by specific glial subtypes, oligodendrocyte and Schwann cells, which enwrap axons with myelin, a multilayered compacted lipid-rich membrane stack [3], [4]. Even though *Drosophila* glia do not generate myelin, specialized glia that ensheath individual axons or fascicles are present in flies [1], [5].

The vital importance of glia in different organisms was clearly illustrated in cell ablation experiments. For example, when oligodendrocytes are ablated in mice, the animals become severely paralyzed and die prematurely [6], [7]. Also in *Drosophila*, where glia constitute a minor cell population [8], genetic ablation of glia induces rapid death of the flies [9]. Whereas the evolutionally conserved significance of glia is undisputed, little is known about the vital functions of glia. Here, we aimed at identifying genes in glia that are indispensable for the function of the nervous system.

## Results and Discussion

### A global *in vivo Drosophila* RNAi screen in glia

**Figure 1:**
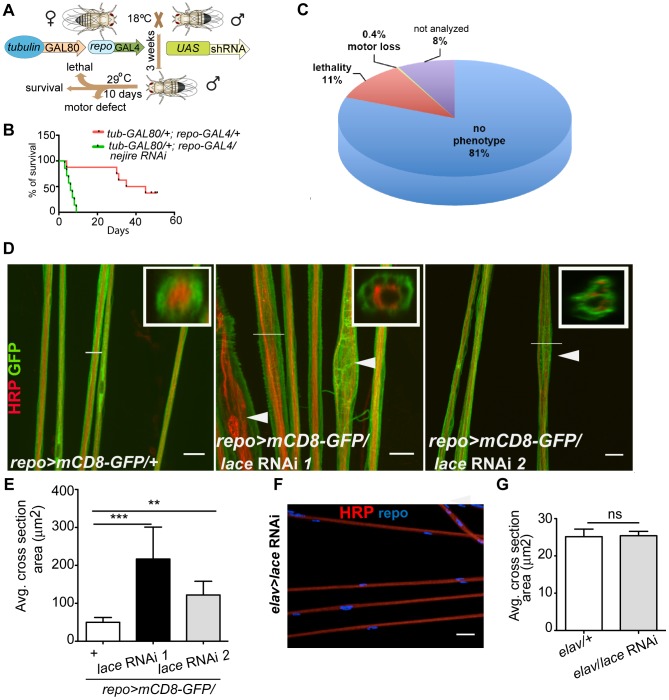
Primary screening and *lace* phenotype. (A) Scheme of the screening strategy. (B) Pre-screening result with *nejire* RNAi with *tub-GAL80^ts^; repo-GAL4*. Survival curves were analyzed with Log-Rank Mantel Cox test. p<0.0001 (C) Results from the primary screening reveal 861 RNAi lines with lethality and 30 RNAi lines with impaired locomotion. (D) Peripheral nerves of L3 larval PNS, glial membrane (green) swelling and axonal (red) wrapping defect were observed upon knockdown of *lace* with 2 different RNAi lines (Transformant ID 21803, 110181). *repo>mCD8-GFP/+* served as a control. Merged projection of confocal z-stacks is presented. In insets, orthogonal sections of the nerve region marked by a white line are shown (E) Quantification of the average cross-section of the peripheral nerves after glial specific knockdown of *lace*. (F) Merged projection of confocal z-stacks did not reveal any visible morphological changes in the neuronal morphology in the PNS after knockdown of *lace* specifically in the neurons (HRP). (G) Quantification of the average cross-section of the peripheral nerves after neuron-specific knockdown of *lace*. For the statistical analysis, unpaired *t-*test was performed with the respective control. Scale bar 20 µm. ns not significant. All graphs represent mean values ± SD. ** p<0.01 *** p<0.001.

In order to identify genes with essential function in glia, we performed a global *in vivo* glial-specific RNAi screening. As we were interested in evolutionary conserved glial functions, we restricted our analysis to all fly genes of which a human ortholog could be identified (as provided by the VDRC) comprising roughly 45% of all protein coding genes in the fly. Therefore, a library of 7881 RNAi lines corresponding to 6930 genes with a putative human ortholog was obtained for the screening [10], [11]. The scheme of the screening is presented in (Figure 1A). The expression of shRNA was restricted to glial cells in adult flies by using the pan-glial driver line *repo-GAL4* [12], [13] in combination with temperature sensitive (ts) GAL80^ts^ under the control of the ubiquitous tubulin promoter (*tub*-*GAL80^ts^*) [14]. Crossing of virgin females (*tub-GAL80^ts^; repo-GAL4*) with 2–3 males from *UAS-shRNA* fly lines were set at 18°C. three to five days post eclosion; male adult flies from F1 generation were shifted to 29°C to induce shRNA expression. After 10 days, RNAi lines showing lethality or climbing deficits (motor defect) in more than 50% of the flies were counted as primary hits. To evaluate the efficiency of the screening system, we performed a pre-screen with selected RNAi lines targeting genes essential for the viability of any cell type. For example, when *UAS-nejire* RNAi transgenic flies were crossed with *tub-GAL80^ts^; repo-GAL4* virgin female flies, a drastic reduction in the lifespan was observed (Figure 1B). Primary screening data indicated that 11% of the total 7881 RNAi lines used in the screening, showed lethality and 0.45% were scored for motor defects (Figure 1C). Since the aim of our primary screen was to identify genes that result in lethality or severe motor defects when knocked down in glia, but not in other cells, we compared our hits with datasets that were obtained by using the same RNAi library but in combination with *GMR-GAL4* (Figure S1). With this approach, we identified 736 candidates with possibly essential functions in glia (Table S1).

Next, by using the list of primary hits and their predicted human ortholog, we performed a systematic networking analysis by Bingo online resource [15] to reveal gene ontology (GO) annotated biological processes. Among the 736 primary hits, we detected 306 genes with a possible function in metabolic processes and among those 79 genes with a predicted function in glial carbohydrate and lipid metabolic processes. Major carbohydrate pathways identified are glycolysis, pentose phosphate and polysaccharide metabolic pathways, while phospholipid, fatty acid and steroid metabolic process were amongst the lipid metabolic pathways. Since metabolic functions are of particular interest in mammalian glia [16], [17], we decided to analyze these hits further in a secondary screen. For the secondary screening, wandering third instar (L3) larval peripheral nervous system (PNS) was chosen because its organization is less complex than that of the central nervous system (CNS). It is easily accessible and renders possible visualization by light and electron microscopy [18], [19]. Moreover, at this stage, the glial migration is complete and the terminally differentiated glia ensheath the afferent and efferent axons of the peripheral nerves [20]–[22]. Axons in each of the peripheral nerve are enwrapped by the wrapping glia, which in turn are encircled by two types of surface glia, the perineurial glia and subperineurial glia [18], [19]. The rational of the secondary screening was to identify the essential metabolic pathway for glial ensheathment of axons. To visualize the glial membrane, a membrane tagged GFP (*UAS-mCD8-GFP*) [23] was expressed using *repo-GAL4* and immunolabeling against HRP was performed to highlight the axons. For the secondary screening, the candidates from lipid and carbohydrate pathways were selected based on STRING protein association database [24] (Figure S2). Secondary screening resulted in various phenotypes such as glial swelling, axonal wrapping defect and axonal splitting in the larval PNS (Figure S3A–C, Table S2) but no embryonic lethality was observed. One of the most penetrant alterations of axon-glia morphology was observed after glia-specific knockdown of lace, a subunit of the serine palmitoyltransferase.

### lace is specifically required in wrapping glia

Serine palmitoyltransferase catalyzes the condensation of serine and palmitoyl-CoA to generate 3-ketosphinganine, the rate-limiting step in *de novo* sphingolipid synthesis [25]. Mutations in the two human subunits of the serine palmitoyltransferase are associated with hereditary sensory and autonomic neuropathy [26]. A common feature of the mutations is the loss of canonical enzyme activity and the generation of toxic lipid intermediates [27]. Glial inhibition of *lace* function (*repo>mCD8-GFP/lace* RNAi) resulted in glial bulging and in an alteration of the axonal packing in all eight pairs of abdominal nerves in all larval PNS examined (n = 15) (Figure 1D). The bulging of glia was localized to focal regions, but appeared randomly along the entire peripheral nerves with diameters ranging from 10 µm to 30 µm. Nerves of *repo-GAL4/+* control flies were straight and packed in bundles with a uniform diameter of 5–8 µm (Figure 1E). In contrast, knockdown of *lace* in neurons did not result in any visible alterations of axonal morphology (Figure 1F). The average cross-section area of the nerve were similar in the knockdown (*elav-GAL4*/*lace* RNAi) and in the *elav-GAL4/+* control flies (Figure 1G)

**Figure 2:**
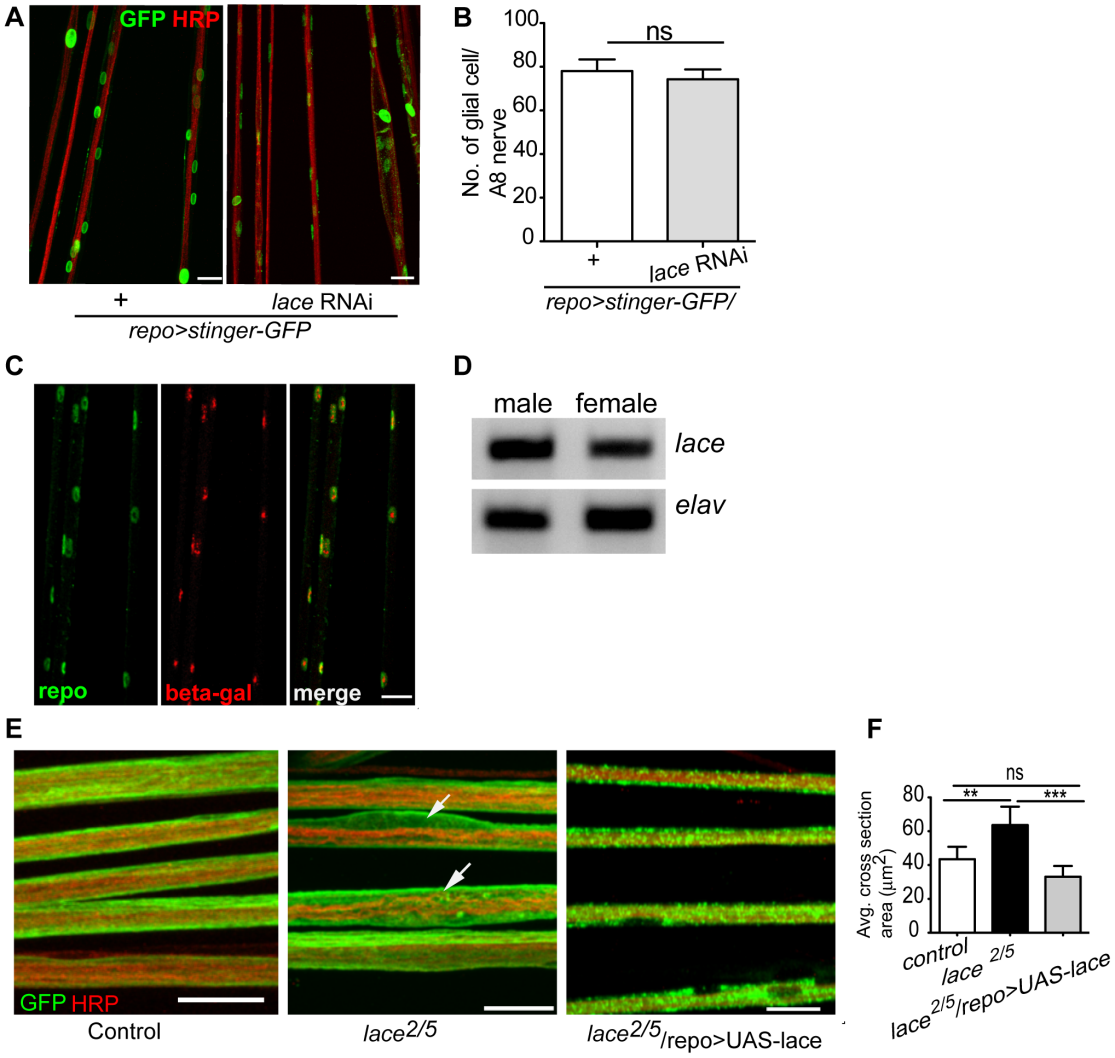
*lace* is expressed in glial cells. (A, B) Quantification of total glial cell number (mean±SD) upon *lace* knockdown in glia. Counting the number of glial nuclei in A8 peripheral nerve (n = 8) revealed no significant differences between control and *lace* RNAi larva. (C) Double immunolabeling of anti-β-galactosidase (red) and anti-repo (green) of *lace^5^* (LacZ enhancer trap line) in L3 larval peripheral nerves. Colocalization shows that lace is expressed in glial cells, present in the peripheral abdominal nerves. (D) RT-PCR analysis showed that lace is expressed in the nervous system of both males and female flies. (E) Hypomorphic combination of *lace* mutant (*lace^2^/lace^5^*) showed axonal defasciculation and increase in the cross-section area of the nerve (arrows). HRP (red) and mCD8-GFP (green) were used to visualize the neuronal and glial morphology, respectively. (F) Quantification showed that the expression of *UAS-lace* by *repo-GAL4* could rescue the mutant phenotype. Scale bar 20 µm. All graphs represent mean values ± SD. Unpaired *t*-test (two groups) and One-way ANOVA followed by Tukey *post hoc* test (for three groups) were performed for the statistical analysis. Scale 20 µm. ** p<0.01 *** p<0.001. ns not significant.

Notably, the ensheathment defect was not due to a compromised blood-nerve-barrier (Figure S4) as has for example been observed in null *fray* mutants [28]. In addition, the number of glial cells in the peripheral nerves was comparable to control (Figure 2A,B). It is important to note glial cell death affects neuronal survival and results in embryonic lethality [9]. The absence of embryonic lethality and the comparable glial cell number suggested that glial cell death did not occur at the larval stage after knockdown of lace. The expression of lace in glia was confirmed by double immunolabeling of *lace^5^* [29] (LacZ enhancer trap line) with anti-β-galactosidase and anti-repo in L3 larval peripheral nerves (Figure 2C). In addition, by RT-PCR analysis of the fly brain and PNS we identified *lace* transcript in the nervous system of both male and female flies (Figure 2D).

Two independent RNAi lines (Transformant ID 21803 and 110181, VDRC) against *lace* showed identical swelling and wrapping defects. In addition, we also observed in hypomorphic *lace* mutant (*lace^2^*/*lace^5^*) axonal defasciculation (Figure 2E), ruling out off-target effects of the RNAi lines. As in the *lace*-RNAi knockdown, the average cross-section area of the nerves was increased in the hypomorphic *lace* mutant animals. The mutant phenotypes appeared to be subtle, which is not surprising as complete loss of *lace* during the development is lethal, while this hypomorphic combination are viable even into adulthood [29]. However, 100% penetrance of the phenotype was observed both for the RNAi knockdown (n = 16) and the hypomorphic mutants (n = 16). Importantly, the *lace* mutant phenotype was rescued by expressing *UAS-lace* specifically in the glial cells (*repo-GAL4*), pointing to an essential function of *lace* in glia (Figure 2E,F).

**Figure 3:**
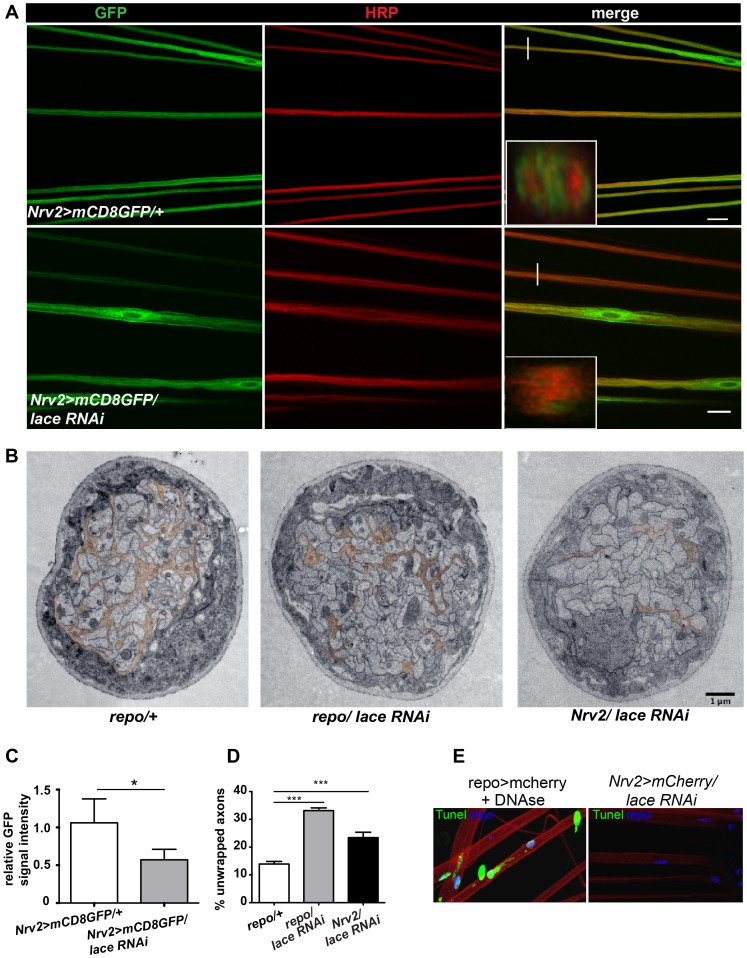
Wrapping glia require lace for axonal ensheathment. (A) *lace* RNAi was expressed specifically in wrapping glia (*Nrv2-GAL4*). mCD8-GFP (green) marks the membrane of wrapping glia and HRP (red) is used to visualize the neuronal membrane. Orthogonal sections from the non-swollen region are presented as insets. Scale 20 µm. (B) TEM micrographs of L3 larval peripheral nerve cross-sections are shown. Wrapping glia is color-coded. Axonal ensheathment is incomplete upon loss of *lace* function in all glia (middle) or in wrapping glia (right). Proper ensheathment of axons is observed only in control (left). Scale 1 µm. (C) Quantification of the GFP signal intensity of wrapping glial membrane was performed on merged confocal projections and *t-*test was used for the analysis. Scale 20 µm.. (D) Quantification of the number of unwrapped axons (8–10 nerves from 4 animals for each genotype). One-way ANOVA followed by Dunnett *post hoc* test was performed. All graphs represent mean values ± SD. * p<0.05 *** p<0.001. (E) TUNEL assay after lace knockdown in the wrapping glia and merged projection of the peripheral nerves are presented. TUNEL (green) positive nuclei are observed only in the positive control (after DNAse addition).

Next, we analyzed, in which of the different glial subtype *lace* was required. Glia subtype specific GAL4 drivers were used to silence *lace* function. A phenotype was only observed when *lace* was depleted in wrapping glia (*Nrv2-GAL4*) (Figure 3A). Quantification of GFP signal intensity revealed that membrane area was significantly reduced as compared to control (*Nrv2>mCD8GFP/lace* RNAi versus *Nrv2>mCD8GFP*) (Figure 3C). Similar results were observed when the *Nrv2>mCD8-mcherry* driver line was used to knockdown *lace* in the wrapping glia (Figure S5 A, E). In contrast, knockdown of *lace* in the two other glial subtypes, the subperineurial (*gliotactin-GAL4*) and the perineurial (*NP6293-GAL4*), did not lead to any visible changes (glial swellings or decrease in the GFP signal intensity) in glia or in axons (–G), suggesting a predominant role of *lace* in the encapsulation of peripheral nerves.

To examine the ultrastructure in more detail we performed electron microscopy. Electron micrographs clearly showed that knockdown of *lace* in glia (*repo/lace* RNAi) severely impaired axonal enwrapping compared to control *(repo/+)* (Figure 3B). Notably, also in the non-swollen regions (A2–A3 segment) of the nerve much less glial processes covered the axons. Quantification demonstrated a significant increase in the number of completely unwrapped axons in this region. We observed a similar phenotype, when *lace* was knocked down in wrapping glia (*Nrv2/lace* RNAi). Again, a clear increase in the completely unwrapped axons was detected as compared to the controls (Figure 3D). Importantly, TUNEL assay could not detect any apoptotic glial nuclei (Nrv2>laceRNAi) suggesting that loss of axonal ensheathment is not because of dying wrapping glial cells (Figure 3E). Together, these results indicate that sphingolipids or intermediates of the sphingolipid pathway are necessary for membrane expansion of wrapping glia.

### Ceramide-phosphoethanolamine is specifically required in wrapping glia

**Figure 4:**
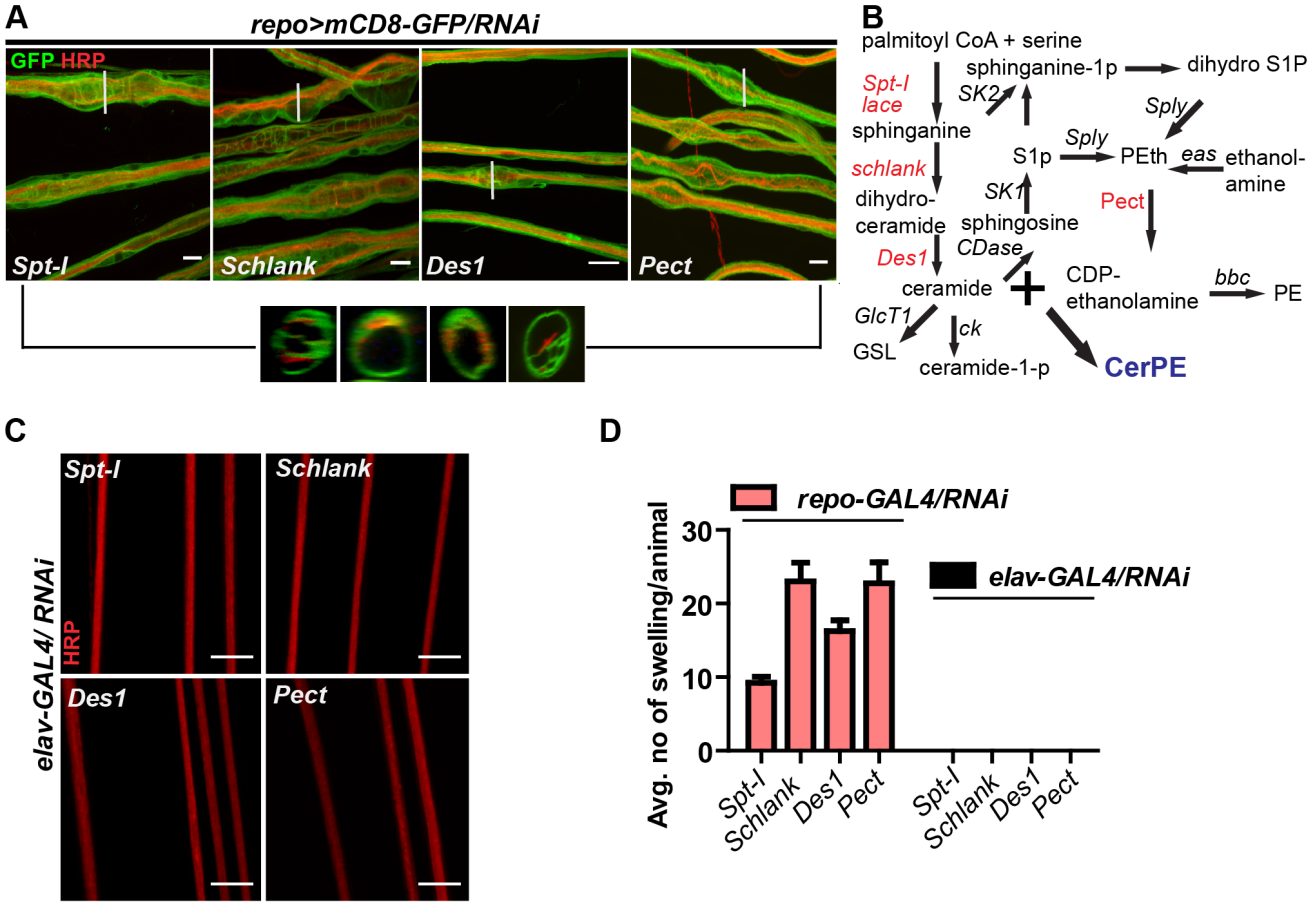
Glia require CerPE for axonal ensheathment. (A) A genetic dissection study of sphingolipid biosynthetic pathway shows that glial knockdown of *Spt-I*, *schlank*, *Des1* and *Pect* result in swelling and wrapping defects (*repo-GAL4*) similar to the *lace* phenotype. Merged projections of confocal stacks are presented. Scale 20 µm. (B) Sphingolipid biosynthesis pathway. (C) RNAi against these 4 genes were expressed in neurons by using *elav-GAL4* driver line. HRP (red) is used to visualize the membrane morphology of the neurons. Scale 20 µm. (D) Quantification (mean± SD) showed that the swellings were only observed upon glial specific knockdown but not in neuron-specific knockdown.

**Figure 5:**
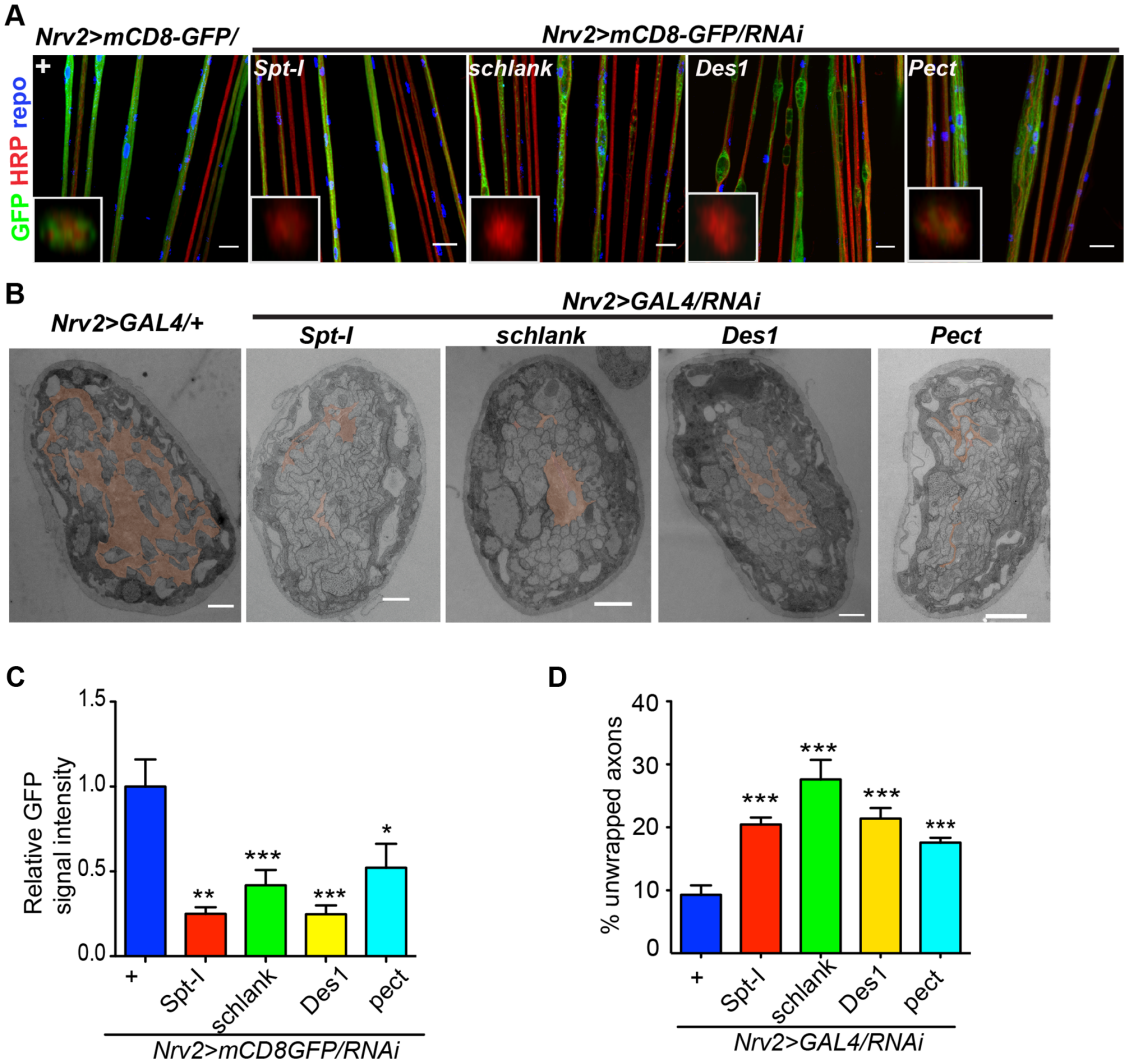
CerPE is very essential for axonal ensheathment by wrapping glia. (A) RNAi against Spt-I, schlank, Des1 and Pect were expressed in wrapping glia (Nrv2-GAL4). Merged projections of the peripheral nerves along with an orthogonal projection are presented. GFP (green) was used to label the wrapping glial membrane, and HRP (red) and repo (blue) were used to label the neuronal membrane and glial nuclei, respectively. (B) TEM micrographs of L3 larval peripheral nerve cross-sections are shown. Wrapping glia is color-coded. Axonal ensheathment is incomplete upon loss of lace function in wrapping glia. Proper ensheathment of axons is observed only in control (+). Scale 1 mm. (C) Quantification of GFP signal intensity of wrapping glial membrane showed a reduction of GFP signal intensity. (D) Quantification of the number of unwrapped axons. One-way ANOVA followed by Dunnett post hoc test was performed. All graphs represent mean values 6 SD. * p,0.05, ** p,0.01, *** p,0.001.

In order to search for the specific sphingolipid (SL) species required by the wrapping glia to mediate axonal ensheathment, a genetic dissection study was performed by expressing RNAi against all known SL metabolic enzymes selectively in glia [30]. Out of 12 genes, we found that knockdown of *Spt-I* [31], *schlank* [32], *Des1* [33] and *Pect* [34] in glia (*repo>mCD8-GFP/*RNAi) with two different RNAi lines (except Des1 due to unavailability) phenocopied the glial swelling and axonal defasciculation as observed upon loss of *lace* function (Figure 4A, S6). Interestingly, all four genes that show 100% penetrance (Table S3) are known to be involved in the biosynthesis of ceramide-phosphoethanolamine (CerPE) (Figure 4B). The specificity of the effect was demonstrated by the absence of any visible phenotype after neuronal specific knockdown of *Spt-I*, *schlank*, *Des1* and *Pect*. (Figure 4C,D). Additionally, glial specific knockdown of different ceramide derivative synthesizing enzymes (*GlcT1*, *CGT*, *CerK*) [35], [36] and PE synthesizing enzyme (*bbc*) [34] did not show any visible defects of axon or glial morphology (Figure S7). Moreover, when *Spt-I*, *schlank*, *Des1* or *Pect* were knocked down specifically in wrapping glia, wrapping defects similar to the *lace* phenotype were observed (Figure 5A). The quantification revealed that the GFP signal intensity was significantly reduced in all four experiments as observed after *lace* knockdown (Figure 5C,S8). Ultrastructural analysis by transmission electron microscopy also showed that wrapping glia failed to extend their membrane around the axons (Figure 5B); and consequently there was an increase of the completely unwrapped axons (Figure 5D). Hence, our data strongly suggests an essential function of glial CerPE in axonal ensheathment by wrapping glia.

**Figure 6:**
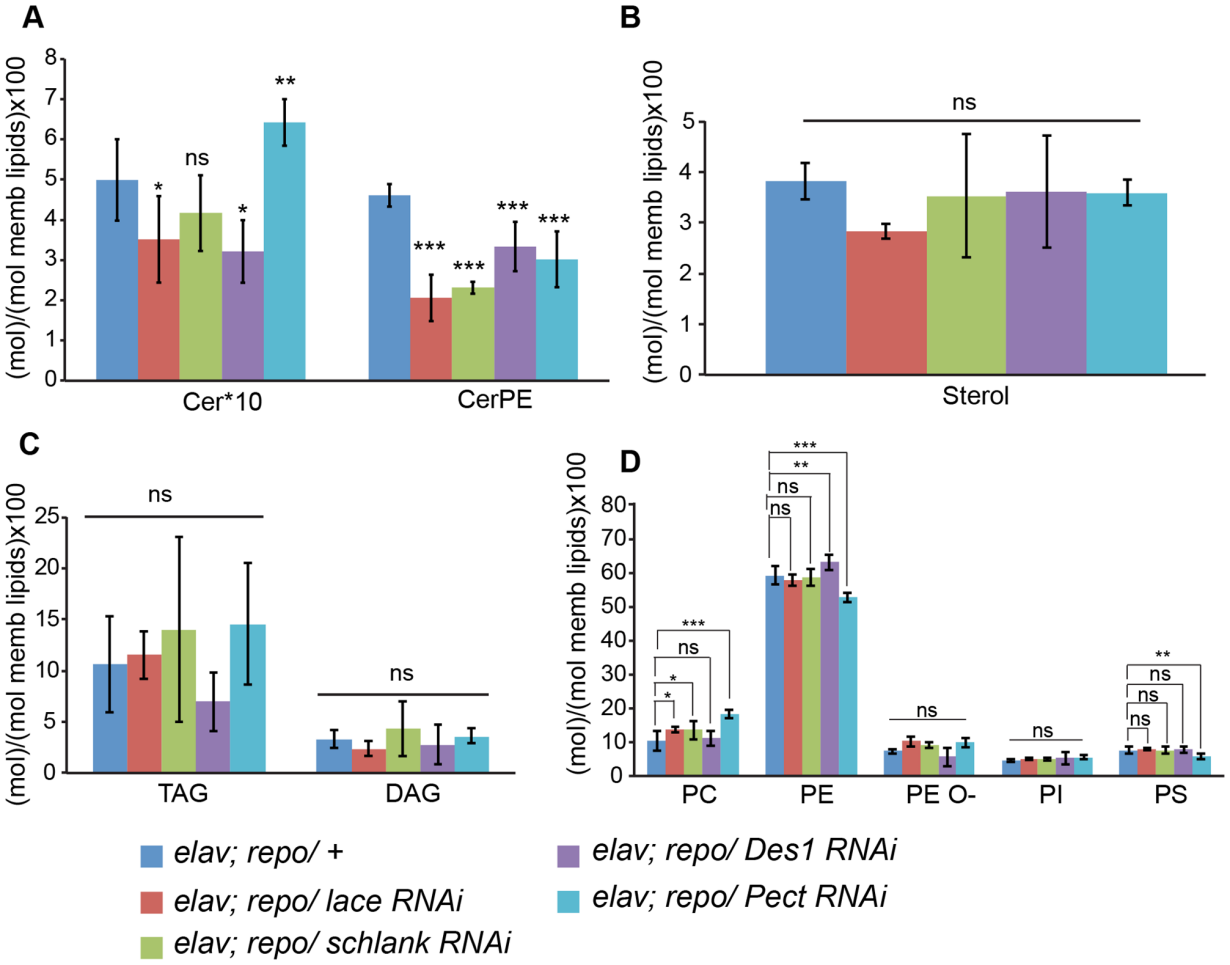
Lipidomics analysis. Lipidomics analysis using high-resolution shotgun mass spectrometry of dissected brains and peripheral nerves derived from L3 larvae. *lace*, *schlank*, *Des1* and *Pect* were downregulated in both neuron and glia, L3 larval brain and peripheral nerves were dissected and the amount of sphingolipid (A), sterol (B), neutral lipid (C) and phospholipids (D) were determined. One-way ANOVA with Dunnett *post hoc* test was used for the statistical analysis. All graphs represent mean values ± SD. * p<0.05 ** p<0.01 *** p<0.001.

In order to analyze whether knockdown of *lace*, *schlank*, *Des1* and *Pect* resulted in depletion of CerPE levels, we performed a detailed lipidomics analysis of the nervous system. This is particularly important in RNAi studies targeting enzymes, because residual enzyme activity due to inefficient RNAi-silencing is often sufficient for their function. Since the nervous system of *Drosophila* only contains 10% of glia, we expressed the RNAi both in neurons and glia using *repo-GAL4* and *elav-GAL4* drivers to deplete the enzymes in the entire nervous system. L3 larval brain and peripheral nerves were dissected and lipidomics analysis was performed with high-resolution shotgun mass spectrometry [37]. Importantly, our lipidomics analysis confirmed that knockdown of *lace*, *schlank*, *Des1* and *Pect* reduced CerPE levels significantly, whereas triacylglycerol (TAG) and diacylglycerol (DAG) and sterol levels were unaltered. Ceramide levels were reduced upon downregulation of *lace* and *Des1*, whereas knockdown of *Pect* lead to increased ceramide levels consistent with its function as a phosphoethanolamine cytidylyltransferase. Phosphatidylcholines (PC) and Phosphatidylethanolamine (PE) levels were slightly changed possibly due to compensatory mechanisms (Figure 6A–D).

**Figure 7:**
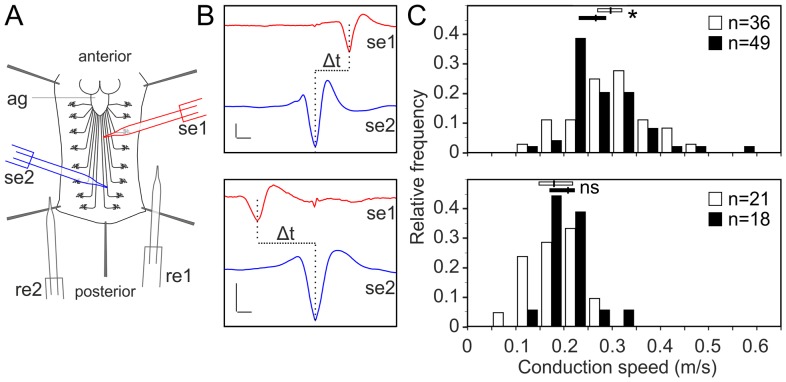
Glial inhibition of *lace* delays afferent spike propagation. (A) Scheme of thoracic and abdominal parts of a dissected larva, electrodes not to scale; ag abdominal ganglion mass, se1, re1 anterior suction electrode and reference electrode, se2, re2 posterior suction electrode and reference electrode. (B) Afferent unit (top) and efferent unit (bottom) recorded simultaneously in 7th right nerve of a *repo>mCD8-GFP/+* control as sketched in (A). Spike templates were generated for se2 and served as trigger events for averaging both se1 and se2. Δt conduction time; bars 50 µV and 1 ms, respectively. (C) Distribution of conduction speed recorded in *repo>mCD8-GFP/+* (white bars) and *repo>mCD8-GFP/lace* RNAi (black bars) animals from afferent (top) and efferent (bottom) units. Horizontal lines indicate medians and their 95% confidence interval of the respective distributions. Difference of medians: * p<0.05 (two-tailed), ns not significant. n number of neurons, recorded from 20 mutant and 9 control larvae.

To test the functional consequences of l*ace* downregulation, we performed paired electrode recordings from abdominal nerves (Figure 7A) and determined the spike propagation velocities of afferent and efferent units (Figure 7B). We found that afferent spike propagation velocities are mildly decreased in *repo>mCD8-GFP/lace* RNAi mutants compared to *repo>mCD8-GFP/+* controls (median velocity smaller by 10.4%), whereas efferent spike propagation velocities remain unchanged (Figure 7C). The apparent reduction of afferent spike propagation velocities was confirmed by bootstrapping (Figure S9), which revealed that the medians of the velocity distributions obtained for *lace* RNAi flies and controls are significantly distinct (p<0.05 (two-tailed), for efferent units p>0.4).

**Figure 8:**
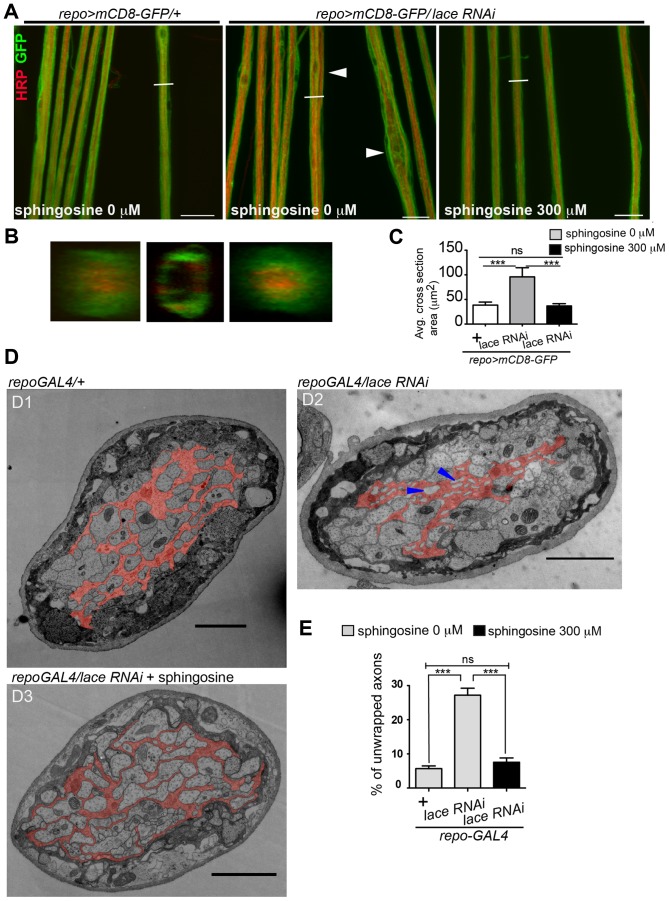
Sphingosine rescues the glial bulging phenotype. (A) Addition of sphingosine to diet rescues the *lace* phenotype. Arrowheads indicate glial swelling region. (B) Orthogonal projections showed that dietary addition of sphingosine rescues the enwrapment defect. (C) Quantification of average cross-section area after knockdown of *lace* in glia and upon sphingosine treatment is shown. All graphs represent mean values ± SD. One-way ANOVA followed by Tukey *post hoc* test was performed for statistical analysis. Scale 20 µm (D1–D3) TEM micrographs of L3 larval peripheral nerve cross-sections are shown. Wrapping glia is color-coded. Vacuoles (blue arrowhead) are present in the wrapping glia and there is loss of membrane extension resulting in lack of proper ensheathement in *repo-GAL4*/*lace* RNAi flies as compared to control *repo-GAL4/+* flies. (D3) Addition of 300 µM sphingosine to the diet (Matreya, USA) rescues the ensheathment defect. (E) Quantification of the number of unwrapped axons before and after sphingosine treatment in the in *lace* knocked down flies. (repo-GAL4/laceRNAi). One-way ANOVA followed by Tukey *post hoc* test was performed. All graphs represent mean values ±SD. *** p<0.001.

Next, we tested whether it was possible to rescue the morphological phenotype induced by knockdown of *lace* in glia by supplementing sphingosine (re-converted to ceramide by condensation with a fatty-acylCoA catalyzed by the various ceramide synthases) into the diet of the flies. Indeed, the phenotype of glia-specific knockdown of *lace* was efficiently rescued by the exogenous addition of sphingosine (300 µM) to the food (Figure 8). Double Immunolabelling of glia and neuronal membrane reveals that the glial bulging and axonal unpacking was rescued upon addition of sphingosine to the diet (Figure 8A). Orthogonal projections (Figure 8B) and the quantification demonstrated the rescue of the neuropathy like phenotype in flies (Figure 8C). We, furthermore, observed with the ultrastructural analysis that the glial enwrapment defect was recovered upon sphingosine addition to the diet (Figure 8D–E). Quantitative analysis of the peripheral nerves using the confocal and electron microscopy showed that the oral administration of sphingosine can restore the enwrapping defect and the neuropathy-like phenotype (Figure 8C,E).

Sphingolipids have both structural and signalling functions in cells. CerPE is a relatively low abundant lipid constituting only around 1% of the total fly lipidome. Interestingly, CerPE appears to be enriched in the fly brain membrane lipidome (4%) (Figure 4A) [37], [38]. In mammals, CerPE is only found in trace amounts, since sphingolipids are in general built on ceramide phosphatidylcholine in higher organisms. There are different possibilities of how CerPE could exert its function in glia. CerPE might be required for signal transduction pathways that control membrane synthesis in wrapping glia. Recently, a mutation in egghead, an enzyme that extends the glycosphingolipids (GSLs) in flies, causes the proliferation and overgrowth of subperineurial glia mediated by aberrant activation of phosphatidylinositol 3-kinase-Akt pathway [39]. CerPE may also increase the packing density of the lipids in the membrane, thereby helping to build up an efficient barrier for the electrical insulation of the axons. In vertebrates, a related sphingolipid, galactocylceramide, is critical for the formation of an insulating myelin sheath in oligodendrocytes. Galactosylceramide and/or its sulphated form are required for the tight sealing of the glial paranodal membrane to the axon [40]. Interestingly, mice lacking ceramide synthase 2 [41], a vertebrate homolog of *schlank*, have myelination defects.

Alterations of enzyme function or enzyme deficiencies do not only result in a reduction in the amount of an essential product, but can also lead to the accumulation of a toxic intermediate, or the production of a toxic side-product For example, mutations in human serine palmitoyltransferase result in a loss of normal enzyme function causing a shift in the substrate specificity, which increase the accumulation of atypical, toxic lipid products [27]. Thus, gain-of-toxic-function is another possibility of how knockdown of *lace* may cause the axonal ensheathment defects.

Interestingly, supplementing sphingosine to the diet restored the ability of wrapping glia to extend their membrane around axons. How diets affect the distribution of lipids in cells and thereby modulate biological processes will be an important question for future investigations. *Drosophila* is an ideal system to pursue such studies because of the short life span and the powerful genetics, which enable rapid and detailed analysis. In summary, our current study illustrates that a large-scale screen in *Drosophila*, in combination with concomitant morphological and electrophysiological analysis has the potential to dissect the basic mechanisms of neuron-glia communication. Detailed knowledge of neuron-glia interactions is a pre-requirement for the rational design of treatment strategies for neuropathies or other diseases in the future.

## Materials and Methods

### Fly strains

Following lines were used in the study w[*]; P{w[+mC] = nrv2-GAL4.S}3 (in the text referred to as *Nrv2-GAL4*) [42], w[*]; P{rl82-GAL4}/CyO (referred to as *Gliotactin-GAL4*) [43], y[*] w[*]; P{w[+mW.hs] = GawB}Bsg[NP6293]/CyO, P{w[-] = UAS-lacZ.UW14}UW14 (referred to as *NP6293-GAL4*) (DGRC,Japan) [44], UAS-mCD8-GFP, UAS-stinger-GFP (kindly provided by Christian Klämbt), lace^5^,*UAS-lace-HA* (kindly provided by Thomas Hummel) w[1118]; P{w[+mC] = GAL4} repo/TM3, Sb (referred to as *repo-GAL4*), w[*]; P{w[+mC] = tubP-GAL80[ts]}20; TM2/TM6B,Tb (referred to as *tub-GAL80^ts^*), P{w[+mW. hs] = GawB}elav[c155] (referred to as *elav-GAL4*) (Bloomington Stock Center). For the screening, we generated a fly line (w; tub-GAL80^ts^; repo-GAL4/TM3, Sb) referred to as *tub-GAL80^ts^; repo-GAL4* by combining glial specific driver repo-GAL4 with ubiquitously expressed temperature-sensitive allele of GAL80^ts^. The RNAi library with predicted human orthologs was provided by VDRC based on common database (status October 2007). For the secondary screening and further morphological analysis, two different RNAi lines were obtained (GD and KK library). Two *lace* mutant lines Adh[n7] lace[2] cn[1] vg[1]/CyO (referred to as *lace^2^*), y[1] w[67c23]; P{w[+mC] = lacW}[45]lace[k05305]/CyO (referred to as *lace^5^*) (Bloomington Stock Center). Flies were provided with standard cornmeal-agar-yeast food if it is not mentioned otherwise.

### Longevity assay

To deplete the mature glial cells *tub-GAL80^ts^; repo-GAL4* flies were crossed with *UAS-nejire* RNAi, and *OregonR* (negative control). 3–4 days post-eclosion, adult males with the respective combination of GAL4-driver, UAS-transgene and GAL80^ts^ were shifted to 29°C (10–15 flies per vial). Numbers of dead flies were counted daily. After 2–3 days, fresh fly food was provided during the assay. At least 50 flies per genotype were used in the assay. Log Rank Test (Mantel-Cox) was used for statistical significance.

### Immunohistochemistry

*Drosophila* L3 stage larva were dissected in 1× PBS (137 mM NaCl, 12 mM Phosphate, 2.7 mM KCl, pH 7.4) and the PNS fixed with Bouin's fixative solution (HT10132, Sigma-Aldrich, Germany for 3 min. Then the tissue was permeabilized with 1× PBT (0.1% Triton-X in PBS) solution for 15 min. For blocking (1 hour) and antibody dilutions 10% goat serum (G9023, Sigma-Aldrich, Germany) was used. Primary antibodies GFP (A11122, Invitrogen, Germany), anti-HRP-Cy3 (123-165-021,Dianova, Germany), antiHRP-alexa647 (123-605-021, Dianova, Germany), anti-repo (8D12, DSHB, University of Iowa, USA) were used with 1∶1000, 1∶200, 1∶200, 1∶20 dilutions, respectively. Primary antibodies were incubated overnight whereas secondary antibodies anti-rabbit alexa-488 (A-11008), mouse alexa-647 (A-21236, both from Invitrogen, Germany) mouse-Cy3 (115-165-14, Dianova, Germany) were used with 1∶200 dilutions for 2 hours. After washing 3 times with PBT, larval mouth part was removed and fillets were mounted in Vectashield (H-1000, VectorLab, USA).

### Quantification of *lace* phenotype

Approximately 200 µm nerve segments were imaged from A3 or A4 body wall segment. Five nerve widths were measured approximately after every 40 µm along the length of the nerve. Every five measurements of each nerve were considered as an ordered quintuplet (d_1_, d_2_, d_3_, d_4_, d_5_) [46]. These five values were used to estimate average cross-sectional area of the nerve with the following equation:
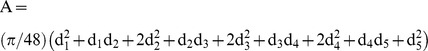



This estimated cross-sectional area of the nerve was calculated by considering the volume of the nerve same as that of a cylinder. At least 5–7 nerves per animals were used to measure this A-value. A-values from each animal were averaged and the mean of these average values were compared between control and *lace* knockdown.

### Quantification of wrapping glia phenotype

For the analysis of wrapping glia defects, *Nrv2-GAL4* line was crossed with different *UAS-shRNA* lines. Images of L3 larval stage PNS were taken for both control and treated groups with exactly the same settings of the confocal microscope (Zeiss, LSM 510). Quantification of the intensity was performed using ImageJ software (NIH, USA). The intensity of the signal was measured as the mean grey value per square micrometer.

### Confocal microscopy

L3 larval PNS was imaged with Zeiss confocal microscope (LSM510) having 40× water-immersion objective. z-stacks images with optical section of 0.5 µm were taken and digital projections of the stack and optical orthogonal section was analyzed using Zeiss LSM image browser software. ImageJ was used for the image processing.

### RT-PCR

*Drosophila* larval brain and PNS was dissected and total RNA was isolated using Macherey Nagel (Germany) RNA isolation kit according to the manufacturer protocol.

1 µg of RNA was used for cDNA synthesis using SuperScript III First-Strand synthesis kit (18080-051, Invitrogen,Germany). 1 µg RNA, 1 µl of 50 µM oligo(dT)20, 1 µl of 10 mM dNTP mix and sterile water were mixed to make up volume to 10 µl. The mixture was incubated at 65°C for 5 min and then cooled down to 4°C. A Reverse Transcriptase mix (RT mix) was prepared by mixing 2 µl 10× RT buffer, 4 µl 25 mM MgCl_2_, 2 µl 0.1 M DTT, 1 µl RNAseOUT and 1 µl Superscript III RT. All of the reagents were provided in kit. RT mix was added to pre-cooled RNA-mix and incubated for 50 min at 50C. The reaction was stopped by increasing the temperature to 85°C for 5 min. 1 µl RNAse H was added and incubated for 20 min at 37°C to cleave remaining RNA. The mixture was cooled to 4°C and cDNA samples were stored at −20°C. cDNA samples were used as template to do a normal semi-quantitative PCR. 2 µl cDNA (1∶10 dilution), 0.3 µl of each primer, 2.5 µl of 25 mM MgCl_2_, 1 µl 10 mM dNTP, 10 µl 5× GoTaq flexi reaction buffer, 0.2 µl of Go-Taq flexi DNA polymerase (M8301, Promega, Germany) were mixed and sterile water was added to make final volume 50 µl. Primers for *elav* are 5′- CGCACAAACCTTATTGTCAACTAC-3′ and 5′-AATTTTACCACTATGGGGTCTGTG-3′. Primers for *lace* are 5′-TTCGACGGCGATTCTGGAAC-3′ and 5′-CAGAGCAATAACCTCGGGCAAA-3′.

### Electron microscopy

For all our experiments, we chose the very late third instar larva namely, wandering L3 larva that stopped eating and climbed away from the food. All larva for the analysis were collected 6 days after egg laying at 25°C. Larval fillets were fixed with a mixture of 4% paraformaldehyde and 2.5% glutaraldehyde in 0.1 M PBS for 4 hours at room temperature. The fillets were washed with PBS and then subjected to post-fixation with 1% osmium tetroxide for 1 hour at 4°C. Next, the post-fixed fillets were dehydrated and stained with a mixture of freshly prepared 1.5% uranyl acetate and 1.5% tungstophosphoric acid. After completion of dehydration process, the fillets were embedded in Epon. Then the silver sections (from A2–A3 regions) were cut and contrasted with 4% uranyl acetate followed by 0.3% lead citrate. Multiple sections were cut, contrasted and imaged for every genotype. The sections were imaged with a LEO EM912 Omega electron microscope (Carl Zeiss, Germany) and the digital micrographs were obtained with an on-axis 2048X2048 CCD camera (Proscan GmbH, Germany).

### Spike propagation velocities

Paired suction electrode recordings of spontaneous spiking activity (20°–23°C, HL3 saline [45]) obtained at the same abdominal nerve in fillet dissection were band-pass filtered (100–3000 Hz) and simultaneously sampled at a rate of 20 kHz. Waveform templates generated with Spike II (Cambridge Electronics, UK) were used as triggers for averaging both electrode signals. Electrode tips were placed on approximately the same z-level and photographed to assess their tip-to-tip distance. Action potential speed distributions were compared by bootstrapping using 10000 repetitions to reveal the 95% confidence intervals of the medians in the two experimental groups.

### Lipidomics analysis

L3 larval brain and peripheral nerves were dissected and then processed for lipid isolation and mass-spectrometry analysis as described before [37]. Briefly, for each replicate, 5 brains were homogenized in 150 mM ammonium bicarbonate using a pestle attached to a cordless motor. For each knockdown experiment, samples were collected from the crossing of three different parents.

Sample volume was adjusted to 200 µl. For absolute quantification, internal standards were added to control for lipid-class dependent differences in extraction and ionization. The internal standard mix contained PG 17∶0 and TAG 36∶0, 10 pmol; Cer 17∶0, GalCer 12∶0 and DAG 17∶0, 20 pmol; PA 17∶0, 25 pmol; PS 17∶0 and PC 18∶3, 40 pmol; PI 17∶0, CerPE 12∶0, Chol-D7 and PE 17∶0, 50 pmol. Lipids were extracted using a modified Folch extraction protocol [47]: 265 µl of methanol was added to the aqueous phase and vortexed; 730 µl of chloroform was added and the samples were vortexed for 1 h; after centrifugation, the organic phase was collected and dried under vacuum to avoid lipid oxidation. The whole extraction procedure including sample preparation was performed at 4°C in order to prevent lipid degradation. All lipid standards were purchased from Avanti Polar Lipids (Alabaster, USA). Solvents were purchased from Sigma-Aldrich (Taufkirchen, Germany).

Mass spectrometric analyses were performed on a QExactive instrument (Thermo Fisher Scientific, Germany) equipped with a robotic nanoflow ion source TriVersa NanoMate (Advion BioSciences, Ithaca, USA) using chips with the diameter of spraying nozzles of 4.1 µm. The ion source was controlled by Chipsoft 8.3.1 software. Ionization voltages were +1.25 kV and −0.9 kV in positive and negative modes, respectively; backpressure was set at 0.95 psi in both modes. The temperature of ion transfer capillary was 200°C; tube voltages were 90 V (MS+) and −150 V (MS−). Acquisitions were performed at the mass resolution R*_m_*_/*z* 200_ = 140 000. AGC control was set at 10^6^ ions and maximum injection time was set to 50 ms.

Dried total lipid extracts were re-dissolved in 100 µl of chloroform: methanol 1∶2. For the analysis, 10 µl of samples were loaded onto 96-well plate (Eppendorf, Germany) of the TriVersa NanoMate ion source and sealed with aluminium foil. Each sample was analyzed for 4 min in positive ion mode where PE, PC PC-*O*, TAG, CerPE and DAG were detected and quantified. This was followed by an acquisition in negative ion mode for 5 min where PA, PI, PS, PG, PE, PEO-, Cer, HexCer were detected and quantified.

Sterol quantification method was performed as described elsewhere [37]. Briefly, dried samples were sulfated with sulfur trioxide pyridine complex in pyridine (Sigma-Aldrich, Germany), sonicated and incubated at room temperature. Then barium acetate (Sigma-Aldrich, Germany) was added, samples sonicated and incubated 10 min at room temperature and then 1 hour at 4°C. Sulfated sterols were quantified in MS mode on QExactive mass spectrometer using cholesterol-D7 (Avanti Polar Lipids, USA) as internal standard.

Lipids were identified by LipidXplorer software [48] by matching the *m/z* of their monoisotopic peaks to the corresponding elemental composition constraints. Mass tolerance was 5 p.p.m and intensity threshold was set according to the noise level reported by Xcalibur software (Thermo Scientific, Germany).

### Dye penetration assay

In order to check the integrity of blood nerve barrier, third instar larva were injected with dextran conjugated Rhodamine dye using standard procedure [49], [50]. 10 kD dextran conjugated Rhodamine was injected in the third instar larval abdominal cavity using a FemtoJet express microinjecting device (Eppendorf). For the injections, glass micropipettes prepared with a P-97 pipette puller (Sutter Instrument) from glass tubes (thin wall, 3 inches, 1-mm diameter; World Precision Instruments) were used. Injection was monitored using a dissection microscope (Leica MZ6). The successful injections were monitored using a stereomicroscope (Zeiss).

After the injections, animals were kept in small Petridis with standard fly food so that they can eat and move. 20–30 min post injection live animals were placed under the microscope after a brief cold shock. Confocal images of larval ventral cord and peripheral nerves were acquired using a Zeiss 510 Confocal microscope.

### TUNEL assay

Terminal deoxynucleotidyl transferase- mediated biotinylated UTP nick end labeling (TUNEL) was performed to detect the wrapping glial apoptosis. In situ cell death detection kit (Roche) was used for the assay and was performed according to the manufacturer protocol.

## Supporting Information

Figure S1 Results of GMR screening. (A) Candidates from primary screening were compared when crossed with GMR-GAL4 to express the RNAi specifically in the eye. The screen resulted in lethality in 3.5% of the flies, whereas 12.4% of the RNAi lines showed a morphological alteration of the red eye (rough eye phenotype). 84.1% RNAi lines did not show any visible defects in the red eye morphology. Images of control flies (GMR-GAL4) (B) and different rough eye phenotypes (C) are presented.
(TIF)

Figure S2 STRING protein association network of lipid and carbohydrate metabolism candidates. Based on the prediction of the STRING protein association database, primary hits of lipid and carbohydrate metabolism are analyzed for their predicted functional associations. Based on this networking, hits that are likely to be associated with three or more candidates (red) are chosen for the secondary screening analysis. The nomenclature is given for *Drosophila* hits and their predicted human ortholog.
(TIF)

Figure S3 Phenotypes of the secondary screening with the selected metabolic candidates. *UAS-*RNAi was expressed using pan-glial driver *repo-GAL4* and the effects were visualized in L3 larval PNS. Different phenotypes that are observed are shown in the figure. A detailed description of the phenotypes is presented in Table S2. Glial membrane was imaged by expressing UAS-mCD8-GFP (green). HRP (red) stained neuronal membrane. Merged projection of all z-stacks is presented in the panel. As a control, the driver line was crossed with wild type. Scale bar 20 µm.
(TIF)

Figure S4 Blood-Nerve-Barrier (BNB) was not compromised. (A) 10 kD Dextran-conjugated Rhodamine dye (red) was injected in the L3 larva. Confocal z-stack of the peripheral nerves (green) and ventral nerve cord show no penetration of the dye in the nervous system. (B) TEM analysis showed no alteration of septate junction morphology.
(TIF)

Figure S5 Glial bulging phenotype was specific to the wrapping glia. *lace* RNAi was expressed specifically in wrapping glia using two different driver line *Nrv2-GAL4* (A-A′), in subperineurial glia (*gliotactin-GAL4*) (B) and in perineurial glia (*NP6293-GAL4*) (C). For the visualization of wrapping glial membrane, mCD8-mcherry and 6STGFP was expressed with *Nrv2-GAL4* driver while mCD8-GFP was expressed to visualize the subperineurial and perineurial glial membrane. Merged projections of confocal z-stacks are represented. HRP (red) immunolabelling was performed to observe the axonal morphology. (D) Average number of swelling regions were quantified in each knockdown experiment and respective driver lines were used as control (n = 8 for each genotype). Swellings were observed upon knockdown of *lace* only in the wrapping glia. The graph represents the mean values + SD. (E–G) Quantification of signal intensity of mcherry and GFP are shown. mcherry level was significantly reduced upon knockdown of *lace* by *Nrv2-*GAL4 whereas GFP signal intensity was unchanged upon *lace* knockdown in subperineurial (Gli/*lace* RNAi) and perineurial glia (NP6293/*lace* RNAi). 10–12 nerves per animal (n = 8) were imaged for the quantification. Scale 20 µm.
(TIF)

Figure S6 Knockdown of *Spt-I*, *schlank*, *Pect* with a second RNAi using *repo-GAL4*. (A) Projection of all confocal stacks after immunolabeling with GFP and HRP shows glial swelling. (B) Orthogonal section of the nerve region marked in white shows axonal defect (red) and glial membrane (green). Repo labels glial nuclei (blue). Scale bar 20 µm.
(TIF)

Figure S7 Glia-specific knockdown of two essential genes in GSL biosynthesis pathway. *GlcT1* and *CGT*, each with two different RNAi driven by *repo-GAL4*, show no visible changes in glial (green) and axonal (red) morphology. *Ceramide Kinase* (CK) knockdown using *repo-GAL4* is also without any obvious phenotype. Glial specific (*repo-GAL4*) knockdown of *bbc*, an essential gene in PE biosynthesis, with two different RNAi (VDRC) does not result in visible effects on axonal (red) and glial (green) morphology. Scale bar 20 µm.
(TIF)

Figure S8 Knockdown of *Spt-I, schlank, Des1 and Pect* affects axonal wrapping. The analysis of the GFP signal intensity along the peripheral nerve after knockdown of *Spt-I*, *schlank*, *Des1* and *Pect* in wrapping glia (*Nrv2>mCD8GFP/RNAi*) as compared to the control (Nrv2>mCD8GFP/+).
(TIF)

Figure S9 Bootstrap analysis of spike propagation velocity differences after *lace* knockdown. Bootstrap statistics for comparing median afferent (left) and efferent (right) spike propagation velocities between *repo>mCD8-GFP/lace RNAi* mutants and *repo>mCD8-GFP/+* controls. Data were resampled 10000 times to compute differences of medians (median_control_ – median*_laceRNAi_*). Black trace: sorted differences; ordinates are scaled to cover the whole range of resampled differences obtained. Red line: difference of medians obtained from the original data sets. Green line: mean of the 10000 resampled differences of medians. Blue lines: 95% confidence range of the resampled differences (i.e. 250 samples generated smaller differences and 250 samples generated larger differences, respectively). As this confidence range includes zero for efferent axons (right), there is no significant difference of median conduction speed between mutants and controls. By contrast, the confidence range excludes zero for afferent axons (left), signalling a significantly altered conduction speed in sensory cells of mutant larvae. Multiple repetition of this resampling procedure yielded p values between 0.0336 and 0.366 (mean p = 0.0356, for two-tailed test). If the test hypothesis is reformulated (‘is conduction speed reduced in *lace RNAi* specimens compared to controls?’), the one-tailed test rejects the null hypothesis with p<0.02, in spite of the numerically small difference of the median conduction speeds (0.0318 m/s) observed in the recordings from afferent units.
(TIF)

Table S1 List of candidates with glial essential function.
(XLSX)

Table S2 Results of the secondary screening using genes from lipid and carbohydrate metabolic pathways.
(XLSX)

Table S3 Genetic dissection of lace phenotype using sphingolipid biosynthetic enzymes.
(XLS)

## References

[1] Freeman MR, Doherty J (2006) Glial cell biology in Drosophila and vertebrates. Trends Neurosci 29: 82–90. 16377000 10.1016/j.tins.2005.12.002

[2] Barres BA (2008) The mystery and magic of glia: a perspective on their roles in health and disease. Neuron 60: 430–440. 18995817 10.1016/j.neuron.2008.10.013

[3] Sherman DL, Brophy PJ (2005) Mechanisms of axon ensheathment and myelin growth. Nat Rev Neurosci 6: 683–690. 16136172 10.1038/nrn1743

[4] Mirsky R, Jessen KR (1996) Schwann cell development, differentiation and myelination. Curr Opin Neurobiol 6: 89–96. 8794046 10.1016/s0959-4388(96)80013-4

[5] Kretzschmar D, Hasan G, Sharma S, Heisenberg M, Benzer S (1997) The swiss cheese mutant causes glial hyperwrapping and brain degeneration in Drosophila. J Neurosci 17: 7425–7432. 9295388 10.1523/JNEUROSCI.17-19-07425.1997 PMC6573436

[6] Pohl HBF, Porcheri C, Mueggler T, Bachmann LC, Martino G, et al (2011) Genetically induced adult oligodendrocyte cell death is associated with poor myelin clearance, reduced remyelination, and axonal damage. J Neurosci 31: 1069–1080. 21248132 10.1523/JNEUROSCI.5035-10.2011 PMC6632929

[7] Buch T, Heppner FL, Tertilt C, Heinen TJAJ, Kremer M, et al (2005) A Cre-inducible diphtheria toxin receptor mediates cell lineage ablation after toxin administration. Nat Methods 2: 419–426. 15908920 10.1038/nmeth762

[8] Edwards TN, Meinertzhagen IA (2010) The functional organisation of glia in the adult brain of Drosophila and other insects. Progress in neurobiology 90: 471–497. 20109517 10.1016/j.pneurobio.2010.01.001 PMC2847375

[9] Booth GE, Kinrade EF, Hidalgo A (2000) Glia maintain follower neuron survival during Drosophila CNS development. Development 127: 237–244. 10603342 10.1242/dev.127.2.237

[10] Vossfeldt H, Butzlaff M, Prussing K, Ni Charthaigh RA, Karsten P, et al (2012) Large-scale screen for modifiers of ataxin-3-derived polyglutamine-induced toxicity in Drosophila. PLoS One 7: e47452. 23139745 10.1371/journal.pone.0047452 PMC3489908

[11] Dietzl G, Chen D, Schnorrer F, Su K-C, Barinova Y, et al (2007) A genome-wide transgenic RNAi library for conditional gene inactivation in Drosophila. Nature 448: 151–156. 17625558 10.1038/nature05954

[12] Lee BP, Jones BW (2005) Transcriptional regulation of the Drosophila glial gene repo. Mech Dev 122: 849–862. 15939231 10.1016/j.mod.2005.01.002

[13] Sepp KJ, Schulte J, Auld VJ (2001) Peripheral glia direct axon guidance across the CNS/PNS transition zone. Dev Biol 238: 47–63. 11783993 10.1006/dbio.2001.0411

[14] McGuire SE, Le PT, Osborn AJ, Matsumoto K, Davis RL (2003) Spatiotemporal rescue of memory dysfunction in Drosophila. Science 302: 1765–1768. 14657498 10.1126/science.1089035

[15] Maere S, Heymans K, Kuiper M (2005) BiNGO: a Cytoscape plugin to assess overrepresentation of gene ontology categories in biological networks. Bioinformatics 21: 3448–3449. 15972284 10.1093/bioinformatics/bti551

[16] Funfschilling U, Supplie LM, Mahad D, Boretius S, Saab AS, et al (2012) Glycolytic oligodendrocytes maintain myelin and long-term axonal integrity. Nature 485: 517–521. 22622581 10.1038/nature11007 PMC3613737

[17] Lee Y, Morrison BM, Li Y, Lengacher S, Farah MH, et al (2012) Oligodendroglia metabolically support axons and contribute to neurodegeneration. Nature 487: 443–448. 22801498 10.1038/nature11314 PMC3408792

[18] Xie X, Auld VJ (2011) Integrins are necessary for the development and maintenance of the glial layers in the Drosophila peripheral nerve. Development 138: 3813–3822. 21828098 10.1242/dev.064816 PMC3261420

[19] Stork T, Engelen D, Krudewig A, Silies M, Bainton RJ, et al (2008) Organization and function of the blood-brain barrier in Drosophila. J Neurosci 28: 587–597. 18199760 10.1523/JNEUROSCI.4367-07.2008 PMC6670337

[20] Parker RJ, Auld VJ (2004) Signaling in glial development: differentiation migration and axon guidance. Biochem Cell Biol 82: 694–707. 15674437 10.1139/o04-119

[21] Banerjee S, Bhat MA (2008) Glial ensheathment of peripheral axons in Drosophila. J Neurosci Res 86: 1189–1198. 18041093 10.1002/jnr.21574 PMC2830789

[22] Sepp KJ, Schulte J, Auld VJ (2000) Developmental dynamics of peripheral glia in Drosophila melanogaster. Glia 30: 122–133. 10719354 10.1002/(sici)1098-1136(200004)30:2<122::aid-glia2>3.0.co;2-b

[23] Lee T, Luo L (1999) Mosaic analysis with a repressible cell marker for studies of gene function in neuronal morphogenesis. Neuron 22: 451–461. 10197526 10.1016/s0896-6273(00)80701-1

[24] Snel B, Lehmann G, Bork P, Huynen MA (2000) STRING: a web-server to retrieve and display the repeatedly occurring neighbourhood of a gene. Nucleic Acids Res 28: 3442–3444. 10982861 10.1093/nar/28.18.3442 PMC110752

[25] Hanada K (2003) Serine palmitoyltransferase, a key enzyme of sphingolipid metabolism. Biochim Biophys Acta 1632: 16–30. 12782147 10.1016/s1388-1981(03)00059-3

[26] Dawkins JL, Hulme DJ, Brahmbhatt SB, Auer-Grumbach M, Nicholson GA (2001) Mutations in SPTLC1, encoding serine palmitoyltransferase, long chain base subunit-1, cause hereditary sensory neuropathy type I. Nat Genet 27: 309–312. 11242114 10.1038/85879

[27] Rotthier A, Penno A, Rautenstrauss B, Auer-Grumbach M, Stettner GM, et al (2011) Characterization of two mutations in the SPTLC1 subunit of serine palmitoyltransferase associated with hereditary sensory and autonomic neuropathy type I. Hum Mutat 32: E2211–2225. 21618344 10.1002/humu.21481

[28] Leiserson WM, Harkins EW, Keshishian H (2000) Fray, a Drosophila serine/threonine kinase homologous to mammalian PASK, is required for axonal ensheathment. Neuron 28: 793–806. 11163267 10.1016/s0896-6273(00)00154-9

[29] Adachi-Yamada T, Gotoh T, Sugimura I, Tateno M, Nishida Y, et al (1999) De novo synthesis of sphingolipids is required for cell survival by down-regulating c-Jun N-terminal kinase in Drosophila imaginal discs. Mol Cell Biol 19: 7276–7286. 10490662 10.1128/mcb.19.10.7276 PMC84720

[30] Dobrosotskaya IY, Seegmiller AC, Brown MS, Goldstein JL, Rawson RB (2002) Regulation of SREBP processing and membrane lipid production by phospholipids in Drosophila. Science 296: 879–883. 11988566 10.1126/science.1071124

[31] Chintapalli VR, Wang J, Dow JAT (2007) Using FlyAtlas to identify better Drosophila melanogaster models of human disease. Nat Genet 39: 715–720. 17534367 10.1038/ng2049

[32] Bauer R, Voelzmann A, Breiden B, Schepers U, Farwanah H, et al (2009) Schlank, a member of the ceramide synthase family controls growth and body fat in Drosophila. EMBO J 28: 3706–3716. 19834458 10.1038/emboj.2009.305 PMC2790492

[33] Basu J, Li Z (1998) The Des-1 protein, required for central spindle assembly and cytokinesis, is associated with mitochondria along the meiotic spindle apparatus and with the contractile ring during male meiosis in Drosophila melanogaster. Mol Gen Genet 259: 664–673. 9819060 10.1007/s004380050861

[34] Lim H-Y, Wang W, Wessells RJ, Ocorr K, Bodmer R (2011) Phospholipid homeostasis regulates lipid metabolism and cardiac function through SREBP signaling in Drosophila. Genes & Development 25: 189–200. 21245170 10.1101/gad.1992411 PMC3022264

[35] Kohyama-Koganeya A, Sasamura T, Oshima E, Suzuki E, Nishihara S, et al (2004) Drosophila glucosylceramide synthase: a negative regulator of cell death mediated by proapoptotic factors. J Biol Chem 279: 35995–36002. 15210713 10.1074/jbc.M400444200

[36] Dasgupta U, Bamba T, Chiantia S, Karim P, Tayoun ANA, et al (2009) Ceramide kinase regulates phospholipase C and phosphatidylinositol 4, 5, bisphosphate in phototransduction. PNAS 106: 20063–20068. 19892737 10.1073/pnas.0911028106 PMC2785292

[37] Carvalho M, Sampaio JL, Palm W, Brankatschk M, Eaton S, et al (2012) Effects of diet and development on the Drosophila lipidome. Mol Syst Biol 8: 600. 22864382 10.1038/msb.2012.29 PMC3421444

[38] Guan XL, Cestra G, Shui G, Kuhrs A, Schittenhelm RB, et al (2013) Biochemical membrane lipidomics during Drosophila development. Dev Cell 24: 98–111. 23260625 10.1016/j.devcel.2012.11.012

[39] Dahlgaard K, Jung A, Qvortrup K, Clausen H, Kjaerulff O, et al (2012) Neurofibromatosis-like phenotype in Drosophila caused by lack of glucosylceramide extension. PNAS 109: 6987–6992. 22493273 10.1073/pnas.1115453109 PMC3344977

[40] Coetzee T, Fujita N, Dupree J, Shi R, Blight A, et al (1996) Myelination in the absence of galactocerebroside and sulfatide: normal structure with abnormal function and regional instability. Cell 86: 209–219. 8706126 10.1016/s0092-8674(00)80093-8

[41] Imgrund S, Hartmann D, Farwanah H, Eckhardt M, Sandhoff R, et al (2009) Adult ceramide synthase 2 (CERS2)-deficient mice exhibit myelin sheath defects, cerebellar degeneration, and hepatocarcinomas. J Biol Chem 284: 33549–33560. 19801672 10.1074/jbc.M109.031971 PMC2785198

[42] Sun B, Xu P, Salvaterra PM (1999) Dynamic visualization of nervous system in live Drosophila. Proc Natl Acad Sci U S A 96: 10438–10443. 10468627 10.1073/pnas.96.18.10438 PMC17907

[43] Sepp KJ, Auld VJ (1999) Conversion of lacZ enhancer trap lines to GAL4 lines using targeted transposition in Drosophila melanogaster. Genetics 151: 1093–1101. 10049925 10.1093/genetics/151.3.1093 PMC1460539

[44] Awasaki T, Lai SL, Ito K, Lee T (2008) Organization and postembryonic development of glial cells in the adult central brain of Drosophila. J Neurosci 28: 13742–13753. 19091965 10.1523/JNEUROSCI.4844-08.2008 PMC6671902

[45] Stewart BA, Atwood HL, Renger JJ, Wang J, Wu CF (1994) Improved stability of Drosophila larval neuromuscular preparations in haemolymph-like physiological solutions. J Comp Physiol A 175: 179–191. 8071894 10.1007/BF00215114

[46] Leiserson WM, Forbush B, Keshishian H (2011) Drosophila glia use a conserved cotransporter mechanism to regulate extracellular volume. Glia 59: 320–332. 21125654 10.1002/glia.21103 PMC3005002

[47] Carvalho M, Schwudke D, Sampaio JL, Palm W, Riezman I, et al (2010) Survival strategies of a sterol auxotroph. Development 137: 3675–3685. 20940226 10.1242/dev.044560 PMC2964098

[48] Herzog R, Schwudke D, Schuhmann K, Sampaio JL, Bornstein SR, et al (2011) A novel informatics concept for high-throughput shotgun lipidomics based on the molecular fragmentation query language. Genome Biol 12: R8. 21247462 10.1186/gb-2011-12-1-r8 PMC3091306

[49] Bainton RJ, Tsai LT, Schwabe T, DeSalvo M, Gaul U, et al (2005) moody encodes two GPCRs that regulate cocaine behaviors and blood-brain barrier permeability in Drosophila. Cell 123: 145–156. 16213219 10.1016/j.cell.2005.07.029

[50] Schwabe T, Bainton RJ, Fetter RD, Heberlein U, Gaul U (2005) GPCR signaling is required for blood-brain barrier formation in drosophila. Cell 123: 133–144. 16213218 10.1016/j.cell.2005.08.037

