# Supplementary figures and images for: A Global In Vivo Drosophila RNAi Screen Identifies a Key Role of Ceramide Phosphoethanolamine for Glial Ensheathment of Axons
Source: PLoS Genet. 2013 Dec 12;9(12):e1003980. doi: 10.1371/journal.pgen.1003980 (PMC3861124; doi:10.1371/journal.pgen.1003980)

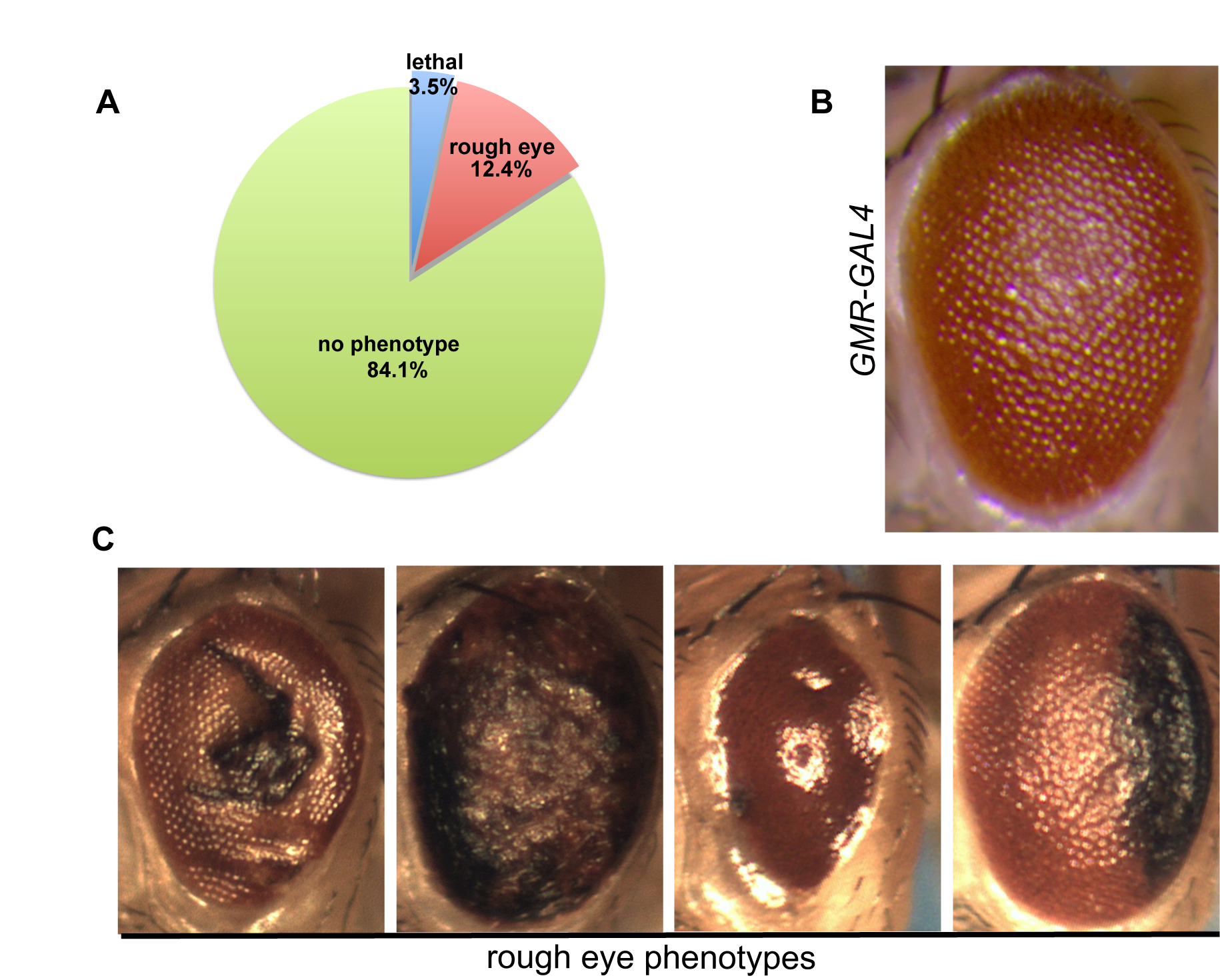

Supplement: Figure S1 — Results of GMR screening. (A) Candidates from primary screening were compared when crossed with GMR-GAL4 to express the RNAi specifically in the eye. The screen resulted in lethality in 3.5% of the flies, whereas 12.4% of the RNAi lines showed a morphological alteration of the red eye (rough eye phenotype). 84.1% RNAi lines did not show any visible defects in the red eye morphology. Images of control flies (GMR-GAL4) (B) and different rough eye phenotypes (C) are presented. (TIF) [file pgen.1003980.s001.tif]

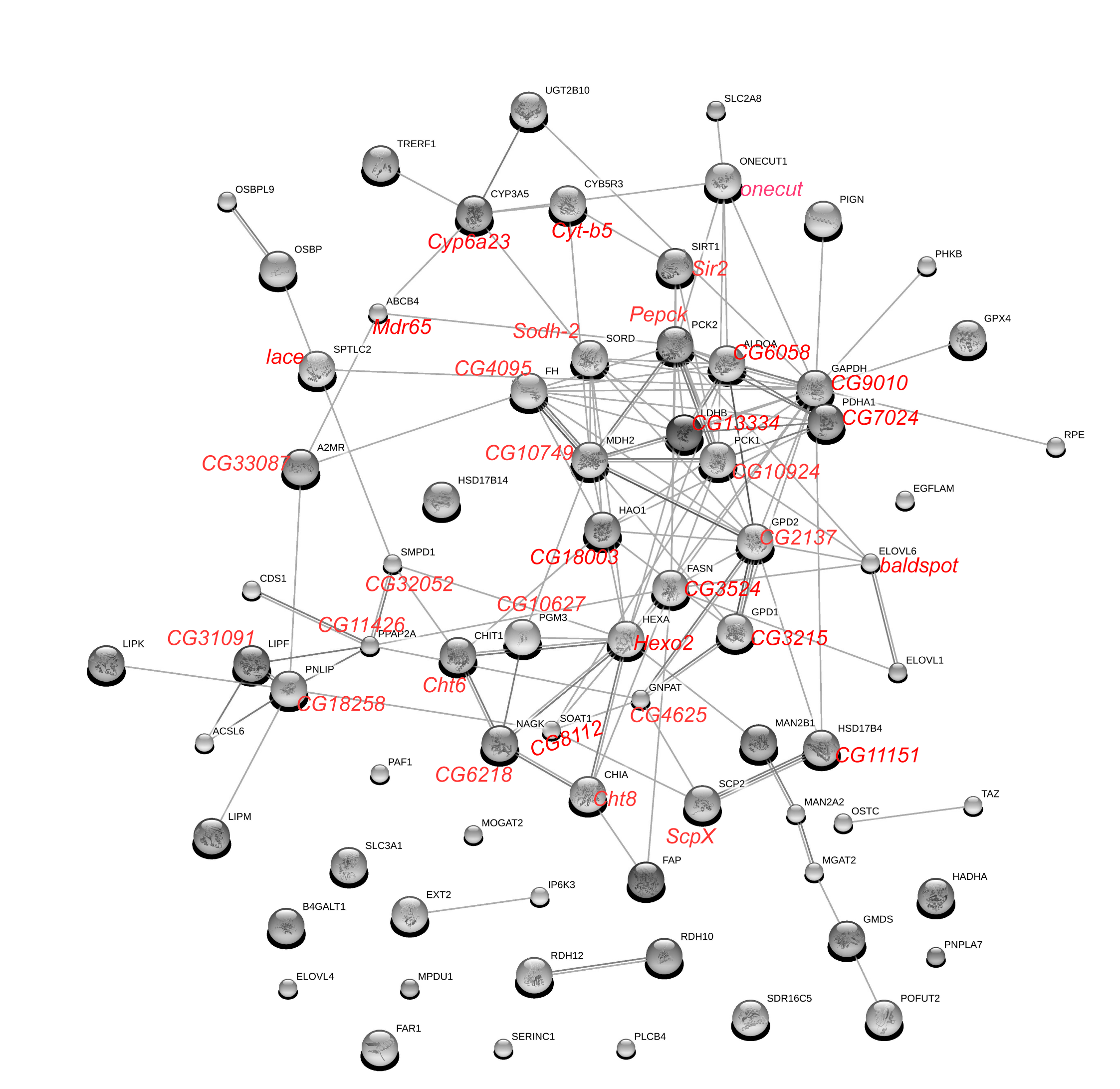

Supplement: Figure S2 — STRING protein association network of lipid and carbohydrate metabolism candidates. Based on the prediction of the STRING protein association database, primary hits of lipid and carbohydrate metabolism are analyzed for their predicted functional associations. Based on this networking, hits that are likely to be associated with three or more candidates (red) are chosen for the secondary screening analysis. The nomenclature is given for Drosophila hits and their predicted human ortholog. (TIF) [file pgen.1003980.s002.tif]

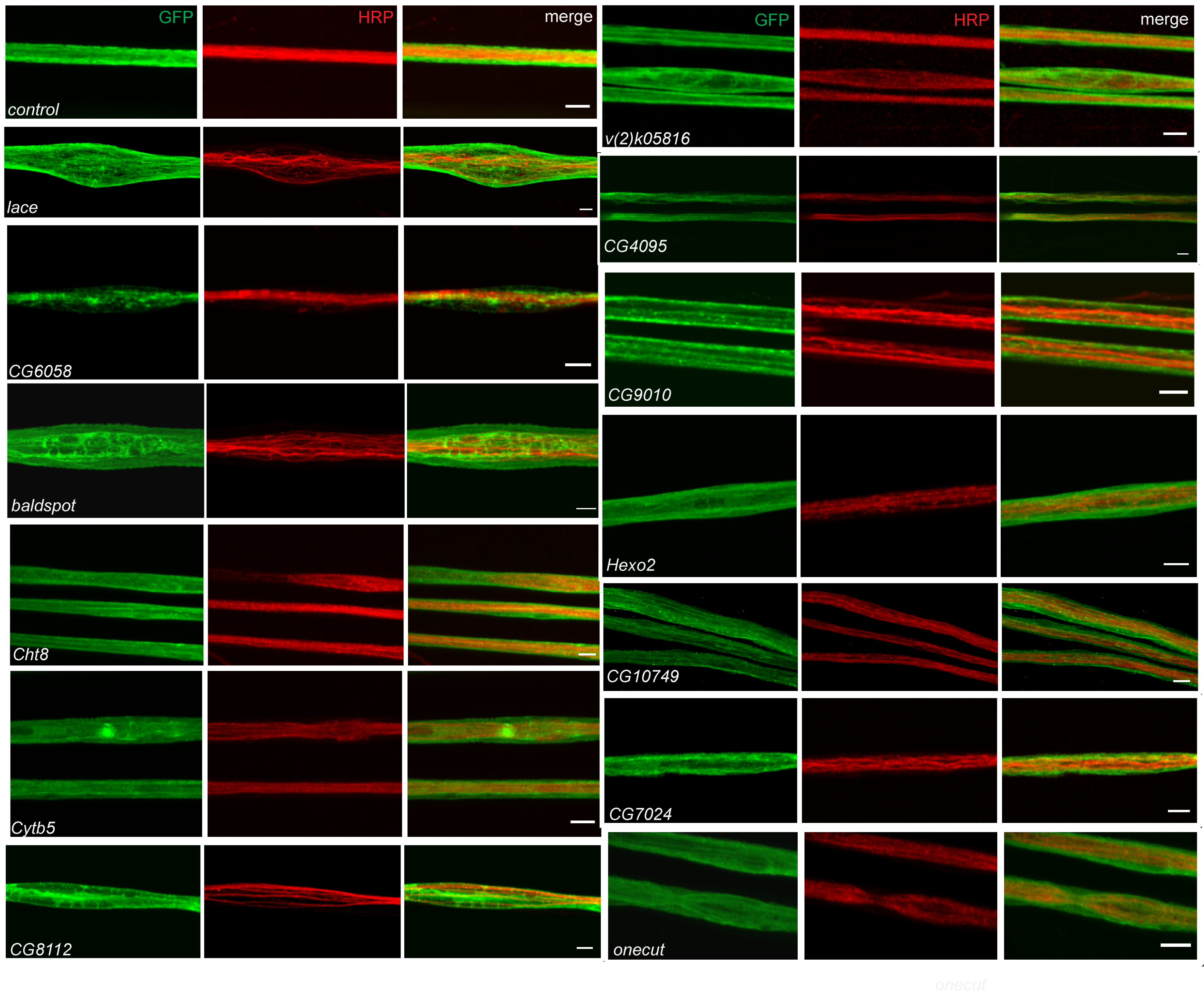

Supplement: Figure S3 — Phenotypes of the secondary screening with the selected metabolic candidates. UAS-RNAi was expressed using pan-glial driver repo-GAL4 and the effects were visualized in L3 larval PNS. Different phenotypes that are observed are shown in the figure. A detailed description of the phenotypes is presented in Table S2. Glial membrane was imaged by expressing UAS-mCD8-GFP (green). HRP (red) stained neuronal membrane. Merged projection of all z-stacks is presented in the panel. As a control, the driver line was crossed with wild type. Scale bar 20 µm. (TIF) [file pgen.1003980.s003.tif]

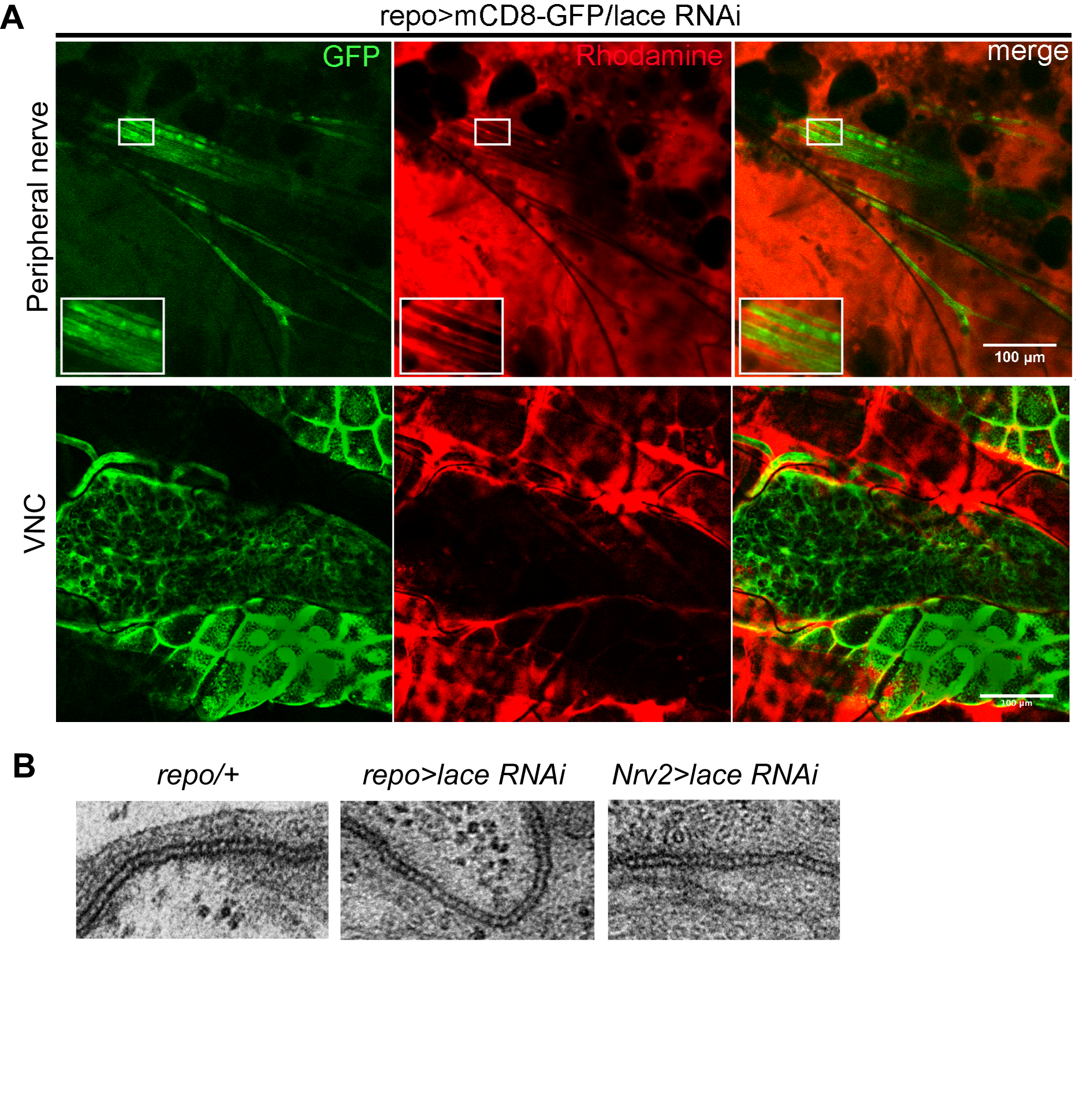

Supplement: Figure S4 — Blood-Nerve-Barrier (BNB) was not compromised. (A) 10 kD Dextran-conjugated Rhodamine dye (red) was injected in the L3 larva. Confocal z-stack of the peripheral nerves (green) and ventral nerve cord show no penetration of the dye in the nervous system. (B) TEM analysis showed no alteration of septate junction morphology. (TIF) [file pgen.1003980.s004.tif]

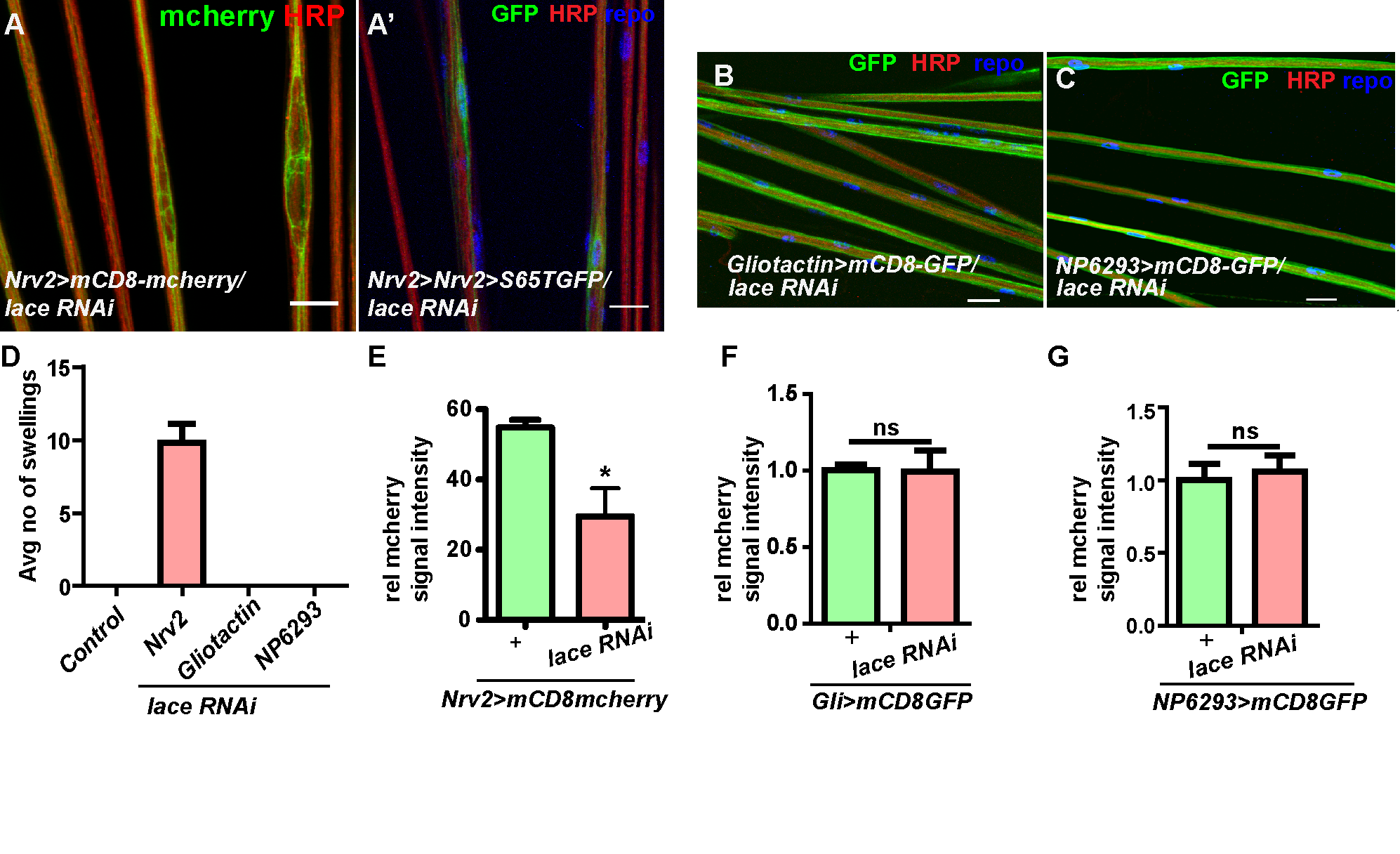

Supplement: Figure S5 — Glial bulging phenotype was specific to the wrapping glia. lace RNAi was expressed specifically in wrapping glia using two different driver line Nrv2-GAL4 (A-A′), in subperineurial glia (gliotactin-GAL4) (B) and in perineurial glia (NP6293-GAL4) (C). For the visualization of wrapping glial membrane, mCD8-mcherry and 6STGFP was expressed with Nrv2-GAL4 driver while mCD8-GFP was expressed to visualize the subperineurial and perineurial glial membrane. Merged projections of confocal z-stacks are represented. HRP (red) immunolabelling was performed to observe the axonal morphology. (D) Average number of swelling regions were quantified in each knockdown experiment and respective driver lines were used as control (n = 8 for each genotype). Swellings were observed upon knockdown of lace only in the wrapping glia. The graph represents the mean values + SD. (E–G) Quantification of signal intensity of mcherry and GFP are shown. mcherry level was significantly reduced upon knockdown of lace by Nrv2-GAL4 whereas GFP signal intensity was unchanged upon lace knockdown in subperineurial (Gli/lace RNAi) and perineurial glia (NP6293/lace RNAi). 10–12 nerves per animal (n = 8) were imaged for the quantification. Scale 20 µm. (TIF) [file pgen.1003980.s005.tif]

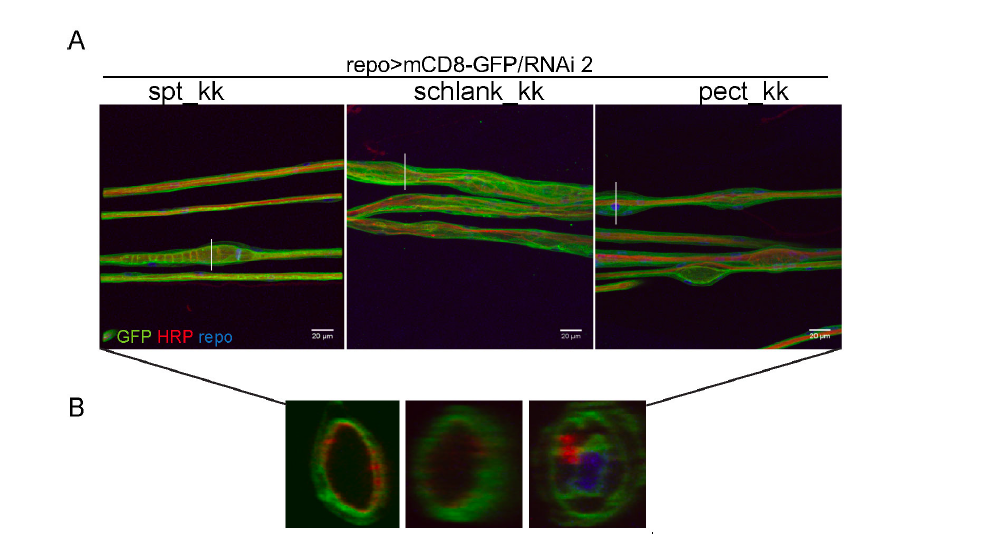

Supplement: Figure S6 — Knockdown of Spt-I, schlank, Pect with a second RNAi using repo-GAL4. (A) Projection of all confocal stacks after immunolabeling with GFP and HRP shows glial swelling. (B) Orthogonal section of the nerve region marked in white shows axonal defect (red) and glial membrane (green). Repo labels glial nuclei (blue). Scale bar 20 µm. (TIF) [file pgen.1003980.s006.tif]

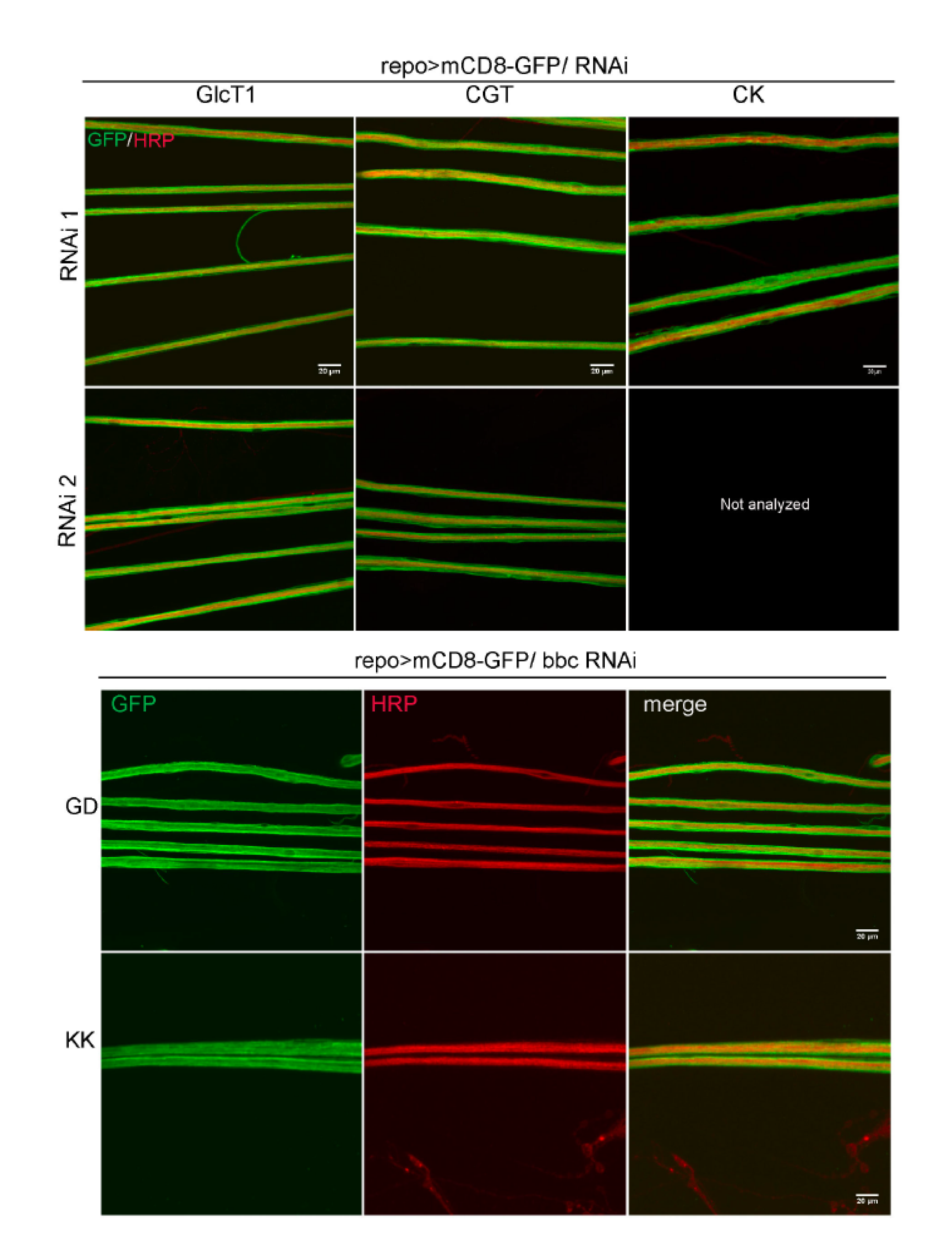

Supplement: Figure S7 — Glia-specific knockdown of two essential genes in GSL biosynthesis pathway. GlcT1 and CGT, each with two different RNAi driven by repo-GAL4, show no visible changes in glial (green) and axonal (red) morphology. Ceramide Kinase (CK) knockdown using repo-GAL4 is also without any obvious phenotype. Glial specific (repo-GAL4) knockdown of bbc, an essential gene in PE biosynthesis, with two different RNAi (VDRC) does not result in visible effects on axonal (red) and glial (green) morphology. Scale bar 20 µm. (TIF) [file pgen.1003980.s007.tif]

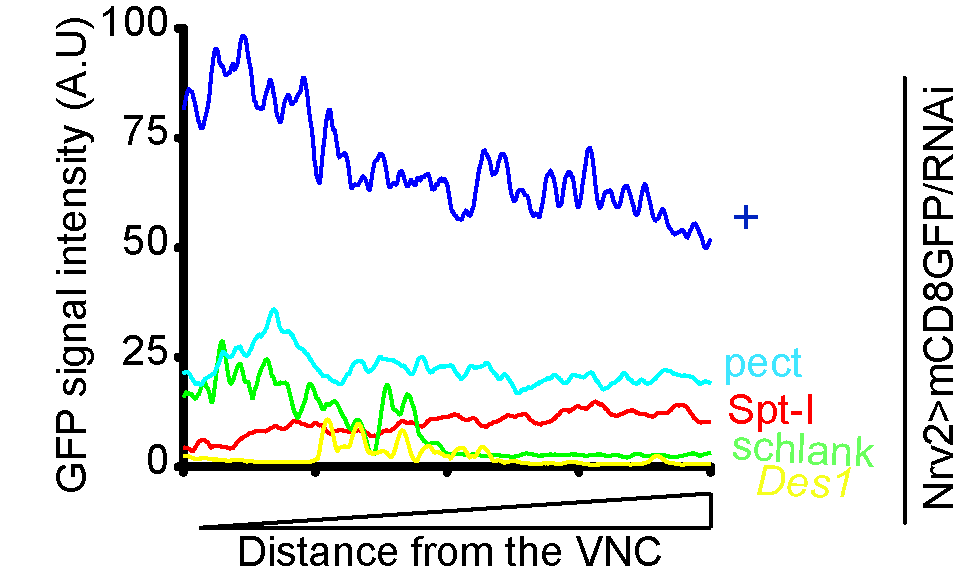

Supplement: Figure S8 — Knockdown of Spt-I, schlank, Des1 and Pect affects axonal wrapping. The analysis of the GFP signal intensity along the peripheral nerve after knockdown of Spt-I, schlank, Des1 and Pect in wrapping glia (Nrv2>mCD8GFP/RNAi) as compared to the control (Nrv2>mCD8GFP/+). (TIF) [file pgen.1003980.s008.tif]

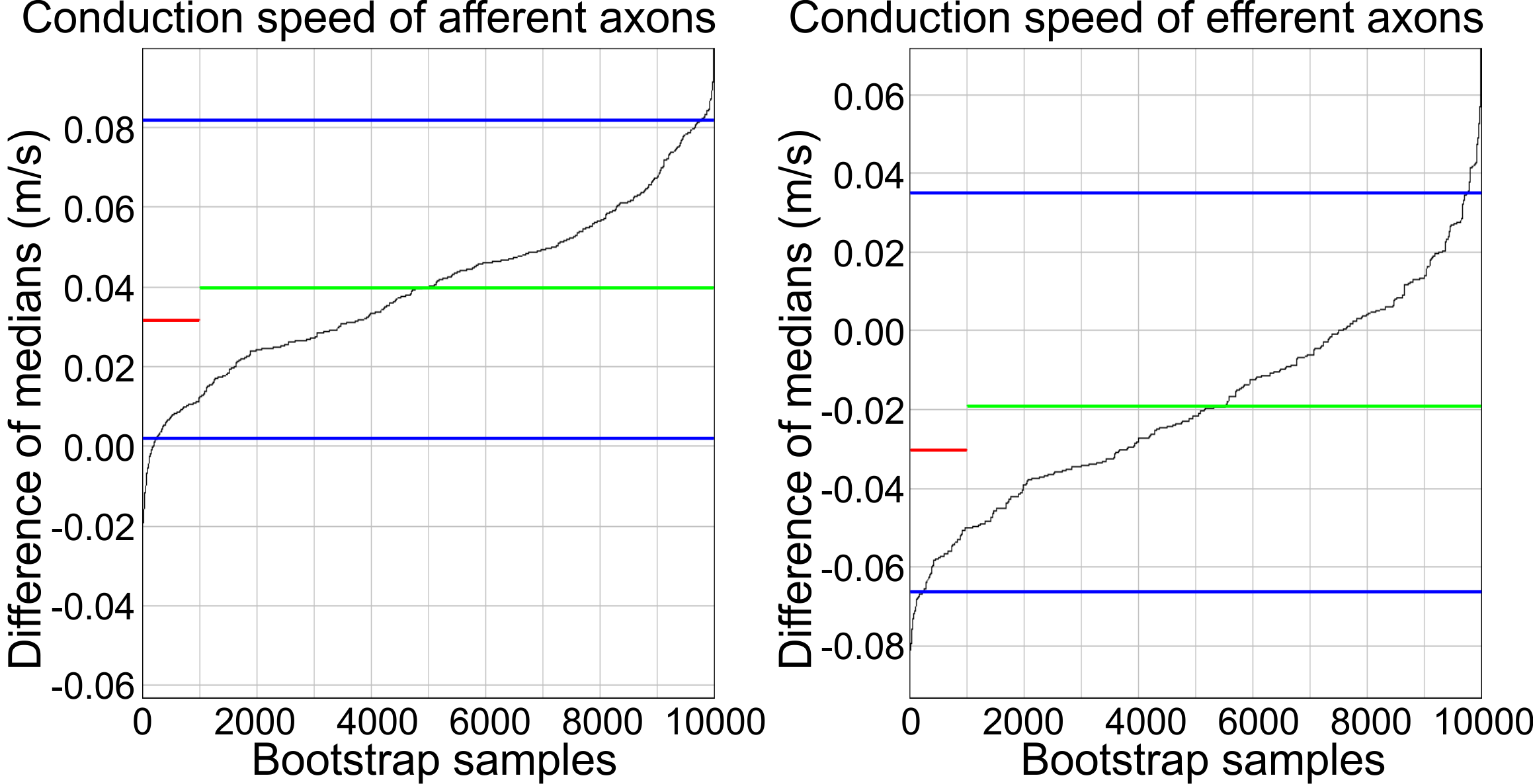

Supplement: Figure S9 — Bootstrap analysis of spike propagation velocity differences after lace knockdown. Bootstrap statistics for comparing median afferent (left) and efferent (right) spike propagation velocities between repo>mCD8-GFP/lace RNAi mutants and repo>mCD8-GFP/+ controls. Data were resampled 10000 times to compute differences of medians (mediancontrol – medianlaceRNAi). Black trace: sorted differences; ordinates are scaled to cover the whole range of resampled differences obtained. Red line: difference of medians obtained from the original data sets. Green line: mean of the 10000 resampled differences of medians. Blue lines: 95% confidence range of the resampled differences (i.e. 250 samples generated smaller differences and 250 samples generated larger differences, respectively). As this confidence range includes zero for efferent axons (right), there is no significant difference of median conduction speed between mutants and controls. By contrast, the confidence range excludes zero for afferent axons (left), signalling a significantly altered conduction speed in sensory cells of mutant larvae. Multiple repetition of this resampling procedure yielded p values between 0.0336 and 0.366 (mean p = 0.0356, for two-tailed test). If the test hypothesis is reformulated (‘is conduction speed reduced in lace RNAi specimens compared to controls?’), the one-tailed test rejects the null hypothesis with p<0.02, in spite of the numerically small difference of the median conduction speeds (0.0318 m/s) observed in the recordings from afferent units. (TIF) [file pgen.1003980.s009.tif]
